# High-density neutrophils in MGUS and multiple myeloma are dysfunctional and immune-suppressive due to increased STAT3 downstream signaling

**DOI:** 10.1038/s41598-020-58859-x

**Published:** 2020-02-06

**Authors:** A. Romano, N. L. Parrinello, V. Simeon, F. Puglisi, P. La Cava, C. Bellofiore, C. Giallongo, G. Camiolo, F. D’Auria, V. Grieco, F. Larocca, A. Barbato, D. Cambria, E. La Spina, D. Tibullo, G. A. Palumbo, C. Conticello, P. Musto, F. Di Raimondo

**Affiliations:** 1 Department of Surgery and Medical Specialties, University of Catania, Catania, Italy; 2 Division of Hematology, Azienda Ospedaliera Policlinico e Vittorio Emanuele di Catania, Catania, Italy; 3 Dipartimento di Scienze Mediche, Chirurgiche e Tecnologie Avanzate “G.F. Ingrassia”, University of Catania, Catania, Italy; 4 Laboratory of Pre-Clinical Research and Advanced Diagnostics, IRCCS-CROB, Rionero in Vulture (Pz), Potenza, Italy; 5 Present Address: Department of Mental Health and Preventive Medicine, Medical Statistics Unit, University of Campania “Luigi Vanvitelli”, Naples, Italy; 6 Biometec, Dipartimento di Scienze Biomediche e Biotecnologiche, University of Catania, Catania, Italy; 7 Present Address: Chair and Unit of Hematology and Stem Cell Transplantation, Aldo Moro University, Bari, Italy

**Keywords:** Translational research, Myeloma

## Abstract

To understand neutrophil impairment in the progression from MGUS through active MM, we investigated the function of mature, high-density neutrophils (HDNs), isolated from peripheral blood. In 7 MM, 3 MGUS and 3 healthy subjects by gene expression profile, we identified a total of 551 upregulated and 343 downregulated genes in MM-HDN, involved in chemokine signaling pathway and FC-gamma receptor mediated phagocytosis conveying in the activation of STAT proteins. In a series of 60 newly diagnosed MM and 30 MGUS patients, by flow-cytometry we found that HDN from MM, and to a lesser extend MGUS, had an up-regulation of the inducible FcγRI (also known as CD64) and a down-regulation of the constitutive FcγRIIIa (also known as CD16) together with a reduced phagocytic activity and oxidative burst, associated to increased immune-suppression that could be reverted by arginase inhibitors in co-culture with lymphocytes. In 43 consecutive newly-diagnosed MM patients, who received first-line treatment based on bortezomib, thalidomide and dexamethasone, high CD64 could identify at diagnosis patients with inferior median overall survival (39.5 versus 86.7 months, p = 0.04). Thus, HDNs are significantly different among healthy, MGUS and MM subjects. In both MGUS and MM neutrophils may play a role in supporting both the increased susceptibility to infection and the immunological dysfunction that leads to tumor progression.

## Introduction

In multiple myeloma (MM), the second most frequent hematological neoplasia, the presence of malignant plasma cells within the bone marrow (BM) is associated to CRAB symptoms including hypercalcemia, renal impairment, anemia and osteolytic bone lesions^1^. Virtually, all MM cases are preceded by an asymptomatic phase defined as monoclonal gammopathy of unknown significance (MGUS) or smoldering myeloma (SMM), characterized by progressive accumulation of plasma cells, from less to more than 10% in the BM^2,3^.

Despite a normal absolute count of neutrophils, recurrent bacterial infections due to both gram positive (e.g. *S. aureus, S. pneumoniae*) and gram negative bacteria (e.g *H.Influenzae*, *E. coli*) are common in MM patients, with the highest risk of blood stream bacterial infections in patients with aggressive clinical presentation^4,5^ within six months from diagnosis^5^. This can be due to the concomitant reduction of the uninvolved immunoglobulins (immunoparesis), since both MGUS^6,7^ and MM patients^8^ have low levels of antibodies against *S. aureus, S. pneumoniae*^6,9,10^, but other host factors associated to the biological features of the underlying disease have been proposed^11,12^.

Neutrophils are terminally differentiated cells which provide to eliminate microbial organisms by phagocytosis, release of cytotoxic granules or extracellular traps^13^, but can work as weak antigen-presenting cells regulating antigen-specific T-cell responses, as recently described^14,15^. Revealing the complex immunological dysregulation in MM, the neutrophil-to-lymphocyte ratio at diagnosis or after 100 days from autologous stem cell transplantation can predict outcome in MM patients, even if the novel agents era^16–22^ In cancer, neutrophils can integrate the environmental signals towards an adaptive response to the tumor-associated sterile inflammation, generally associated to a pro-tumoral phenotype^13,23–26^. Defects in neutrophil maturation, including reduced lysozyme activity^27^ and increased expression of arginase^28,29^, have been described in MM as consequence of the infiltration of the bone marrow by neoplastic cells^27^, but little is known about the contribution of human HDNs in progression from MGUS to MM.

In humans, there are two different functional states of neutrophils, originating from the same cell type^30,31^, not distinguishable by immune phenotype but for their different physical properties. By layering peripheral whole blood over a density gradient medium, high-density neutrophils (HDNs) sediment to the bottom in healthy subjects, while low-density neutrophils, (LDNs) co-purify with mononuclear cells to the top of the gradient in cancer patients^32^. It is well-accepted that LDNs include granulocytic-like myeloid-derived suppressor cells (G-MDSC)^23,32,33^, with unique immune-suppressive properties^34^. Both LDNs and HDNs promote tumor growth and chemo-refractoriness, contributing to the MM pathogenesis in mice^33,35,36^. In humans, LDNs, firstly reported in chronic inflammatory disease, induce in cancer T-cell anergy via secretion of aminoacid degrading enzymes arginase and tryptophan^37,38^.

Taking advantage of an unbiased approach based on gene expression profile, we explored the transcriptome of MGUS and MM-HDNs, showing that myeloid dysfunction occurs early in MGUS, and it is largely associated to increased intracellular signaling triggered by extracellular cytokines, chemokines, bacterial peptides and lipopolysaccharide.

## Results

### In comparison with healthy donors, MGUS- and MM-HDNs have unique gene expression profile

To study overall differences and similarities between healthy, MGUS- and MM- HDNs, we performed gene expression profile using Illumina HumanHT-12 v4 bead arrays. We found that gene expression profiles of MM- MGUS- and healthy HDNs clustered separately (Supplementary Fig. 1). The direct pair-wise comparison between MGUS- and healthy- HDNs identified a total of 749 genes, 491 upregulated and 258 downregulated. The direct pair-wise comparison of MM- and healthy- HDNs identified total of 894 differentially expressed genes: 551 upregulated and 343 downregulated, with a false discovery rate <0.05, (Supplementary Table 1). The direct pair-wise comparison between MGUS- and MM- HDNs identified a total of 3182 differentially expressed genes: 1491 upregulated (with 133 genes at least 3-fold upregulated) and 1691 downregulated (with 430 genes at least 3-fold downregulated).

Only 42/551 genes upregulated in MM-HDNs were also up-regulated in MGUS- HDNs, with 5 genes (CSK, GSA, MEGF, PGM1 and PROK2) significantly associated to progression from MGUS through MM (Supplementary Table 2). Similarly, only 43/343 genes downregulated in MM-HDNs were also down-regulated in MGUS-HDNs, with only 3 genes (FRG1, JOSD1 and one still to be identified) significantly associated to progression from MGUS through MM (Supplementary Table 2).

**Figure 1:**
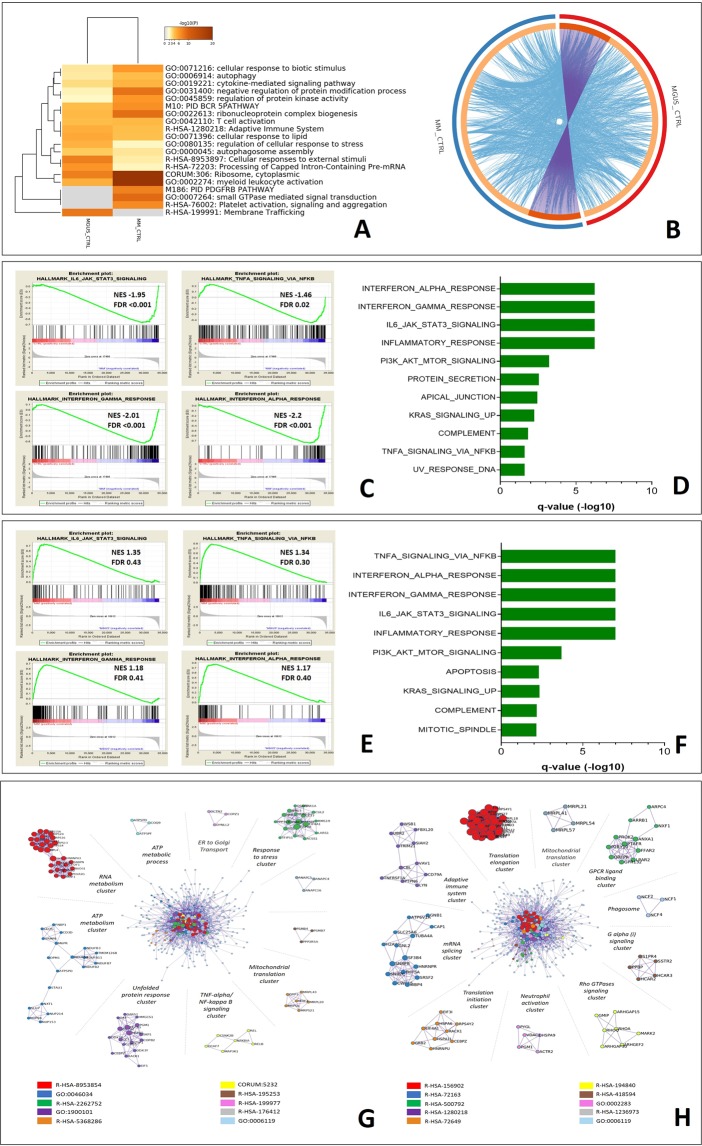
Functional enrichment analysis of high-density neutrophils isolated from multiple myeloma and MGUS patients differ from the control cells at gene level. (**A**) The Metascape suite of tools (http://metscape.org) was used to analyze gene targets differentially expressed in MGUS, MM and healthy HDNs. The color of heatmap depicts statistical enriched terms across input lists of genes significantly enriched (±1.5 fold, p < 0.05) in the comparison of MGUS or MM versus healthy HDNs, white cells: lack of enrichment. (**B**) Circos plot to decipher the overlap between gene lists: where purple curves link identical genes, while blue curves link genes that belong to the same enriched ontology term. In the inner circle each arc represents a gene list, where each gene has a spot on the arc. On the outside, each arc represents the identity of each gene list (MGUS = Red, MM = Blue). (**C**) Gene set enrichment analysis (GSEA) of MM- versus healthy HDNs. Normalized enrichments score (NES) and false discovery rate (FDR) are shown per each gene set analyzed. The green curves show the enrichment score and reflects the degree to which each gene (black vertical lines) is represented at the bottom of the ranked gene list. The heat map indicates the relative abundance (red to blue) of the genes specifically enriched in MM-HDNs as compared with the control cells. (**D**) The top ten gene sets enriched in MM-HDNs cells as compared with the control cells are shown (FDR < 5%). (**E**) GSEA of MM- versus MGUS HDNs with NES and FDR are shown per each gene set analyzed. (**F**) The top ten gene sets enriched in MM-HDNs cells as compared with the MGUS-HDNs are shown (FDR < 5%). (**G**) For the list of genes differentially expressed in MGUS and healthy HDNs, protein-protein interaction enrichment analysis has been carried out with the following databases: BioGrid8, InWeb_IM9, OmniPath and the protein-protein interaction (PPI) network, containing the subset of proteins that form physical interactions with at least one other member in the list, was analyzed. To assign meanings to the network component, GO enrichment analysis was applied to each MCODE component, separated out and aligned radially around the full interactome, identified by a unique color. (**H**) For the list of genes differentially expressed in MM and healthy HDNs, PPI network is shown. Each MCODE component in the merged network was assigned a unique color and has been separated out and aligned radially around the full interactome.

In the attempt to classify the changes in gene ontology, which could suggest characteristic features of HDNs in the progression from MGUS through MM, we identified statistically significant changes in the hallmark gene sets by bioinformatic analysis using Metascape tools (ANOVA p < 0.05; fold difference ±1.5, Fig. 1A). The circos plot depicted in Fig. 1B shows that the total number of genes whose expression was altered in the MGUS set (red) was very distinct from MM setting (blue) when compared to normal.

**Figure 2:**
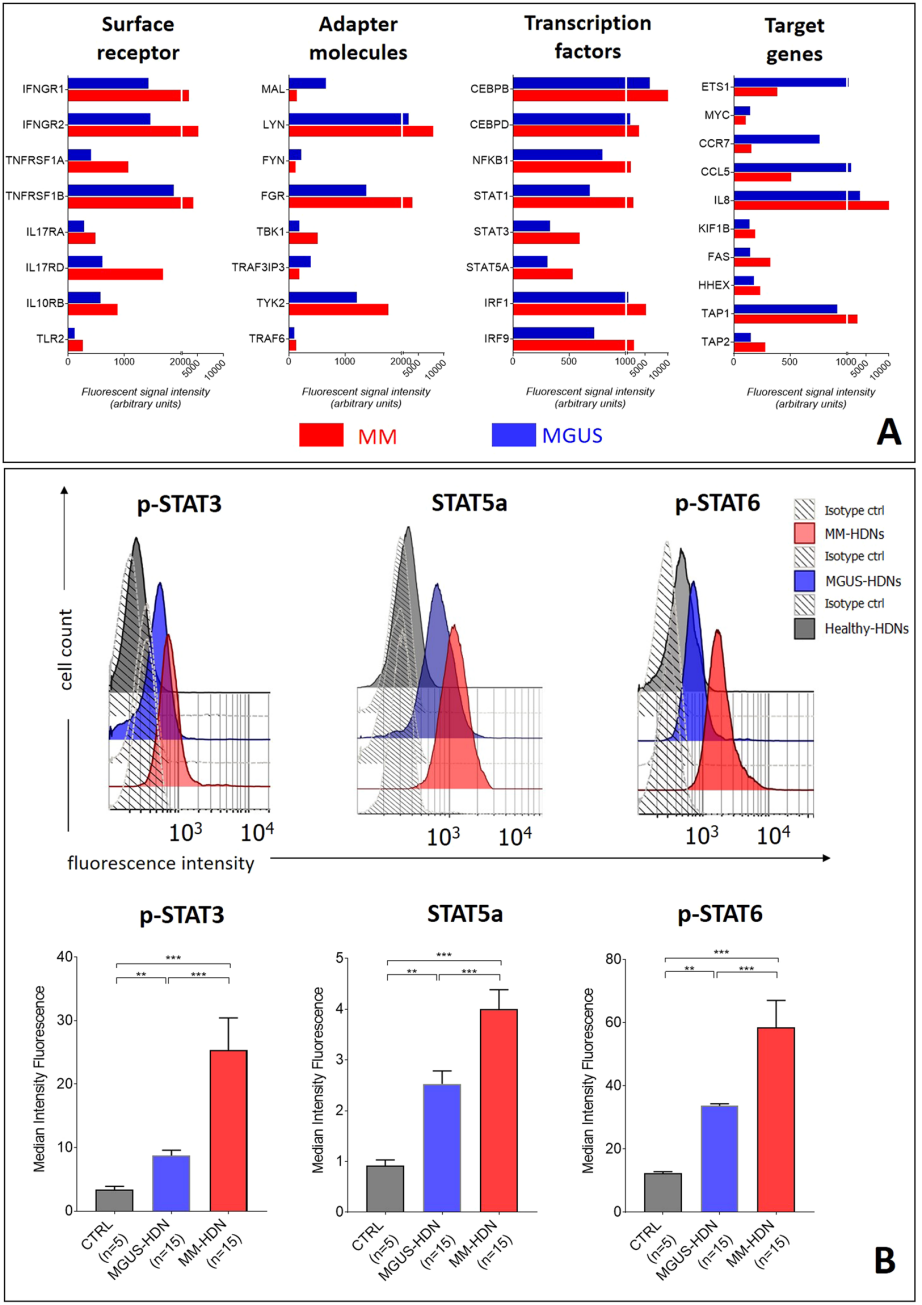
MM-high density neutrophils are chronically activated due to increased STAT signaling. (**A**) Bar charts of genes enriched (FDR < 0.001) in MM-(red) and MGUS-(blue) HDNs, distinguished on their intracellular function, showing the increased signaling via IFN-γ, TNF-α, IL-10-, IL17 and Toll-like receptors. (**B**) To validate the findings of GEP analysis, we tested in an independent set the median of fluorescence intensity (MFI) of STAT3pS727, STAT5a and STAT6pY641 by flow cytometry. For more robust statistical evaluation, MFI values were converted to a resolution metric, such as the RD defined as (Median_treatment_ − Median_control_)/(rSD_treatment_ + rSD_control_) to further perform ANOVA with post-hoc analysis to compare results of different experiments and runs. Stars denote p-value (***p < 0.001).

Using Gene Set Enrichment Analysis (GSEA), we categorized and grouped large gene sets based on a known functional association, derived from the KEGG and HALLMARK pathway databases, generated from publicly available resources (https://www.genome.jp/kegg/pathway.html). This analysis revealed that, compared to both healthy donors (Fig. 2C,D) and MGUS (Fig. 2E,F), MM-HDNs had genes dysregulated in FC-γ-R mediated phagocytosis, endocytosis, leukocyte trans-endothelial migration, chemokine signaling Toll-like receptor pathways and inositol-phosphate metabolism (Table 1).

**Table 1:** Enriched genesets in MM HDNs using GSEA analysis.

MM HDN	SIZE	ES	NES	NOM p-val	FDR q-val	FWER p-val	RANK AT MAX	LEADING EDGE
HALLMARK_INTERFERON_ALPHA_RESPONSE	96	−0.75	−2.20	0.00	<0.0001	<0.0001	3954	tags = 63%, list = 12%, signal = 71%
HALLMARK_INTERFERON_GAMMA_RESPONSE	199	−0.63	−2.01	0.00	<0.0001	<0.0001	3954	tags = 51%, list = 12%, signal = 58%
HALLMARK_IL6_JAK_STAT3_SIGNALING	87	−0.67	−1.95	0.00	<0.0001	<0.0001	4482	tags = 46%, list = 13%, signal = 53%
HALLMARK_INFLAMMATORY_RESPONSE	199	−0.56	−1.80	0.00	<0.0001	<0.0001	4860	tags = 37%, list = 14%, signal = 43%
HALLMARK_PI3K_AKT_MTOR_SIGNALING	104	−0.58	−1.74	0.00	<0.0001	<0.0001	3946	tags = 34%, list = 12%, signal = 38%
HALLMARK_PROTEIN_SECRETION	96	−0.56	−1.66	0.00	<0.0001	0.01	3723	tags = 42%, list = 11%, signal = 47%
HALLMARK_APICAL_JUNCTION	199	−0.51	−1.63	0.00	<0.0001	0.02	4298	tags = 26%, list = 13%, signal = 29%
HALLMARK_MITOTIC_SPINDLE	197	−0.51	−1.62	0.00	<0.0001	0.02	4356	tags = 31%, list = 13%, signal = 35%
HALLMARK_KRAS_SIGNALING_UP	200	−0.50	−1.61	0.00	0.01	0.04	3185	tags = 22%, list = 9%, signal = 24%

Abbreviations: FWER, family-wise error rate; NOM, nominal.

The top enriched genesets as ranked by NES are shown for Hallmark genesets.

Among the list of genes that were clustered in pathway and process enrichment analysis we evaluated the protein-protein interaction (PPI) enrichment. The Metascape tool predicts PPI network by comparing it with protein interaction databases (BioGrid, InWeb_IM, and OmniPath)^39^. The molecular complex detection (MCODE) method was applied to identify closely related protein from PPI network, confirming that in MGUS there was a transcriptional response to stress converging on the TNF-alpha/NfKB signaling (Fig. 1G), while in MM the activation signaling involving GPCR-ligand binding cluster converged in changes in the translational activities (Fig. 1H).

### In MM-HDNs the signaling through IFNγ- and Toll-like receptors is increased to convey chronic inflammatory response via STAT proteins activation

Compared to MGUS, high-expression genes in MM-HDNs were enriched for inflammatory responses to interferon gamma (IFN-γ), lipopolysaccharide (LPS), tumor-necrosis factor alpha (TNFα), interleukins IL-10, IL-17 and IL-6, associated to increased expression of the cognate type II cytokine receptors IFNGR1/2 and IL10RB, TNFα receptors type 1 and 2, the innate immune receptor TLR2 and IL17RA/IL17RD (Fig. 2A), conveying to upregulation of the expression of genes coding for the signal transducer and activator of transcription (STAT) protein family STAT-1, STAT-3, STAT-5 and STAT-6^40–42^ (Supplementary Table 3). Other transcription factors upregulated in MM-HDNs included CEBPβ/CEBPδ and NFKBI, associated to increased expression of their cognate target genes IL-8, FAS, HHEX, TAP1 and TAP2 and reduced expression of ETS1, CCR7 and CCL5, known to be associated to the defective antigen-presentation in cancer^43^.

Activity of the STAT proteins was validated by flow cytometry. In freshly collected sorted primary samples from MM, MGUS and healthy subjects, we found that phosphorylation, at the steady state, of STAT3pS727, STAT6pY641 and the amount of STAT5a were progressively increased from healthy through MGUS – and MM HDNs (ANOVA test respectively, p = 0.002, p = 0.0012 and p = 0.00, Fig. 2B).

### Phagocytosis and oxidative burst are impaired MM- and MGUS- HDNs, despite they are primed by gram-negative bacteria and soluble factors acting upstream of STAT3

**Figure 3:**
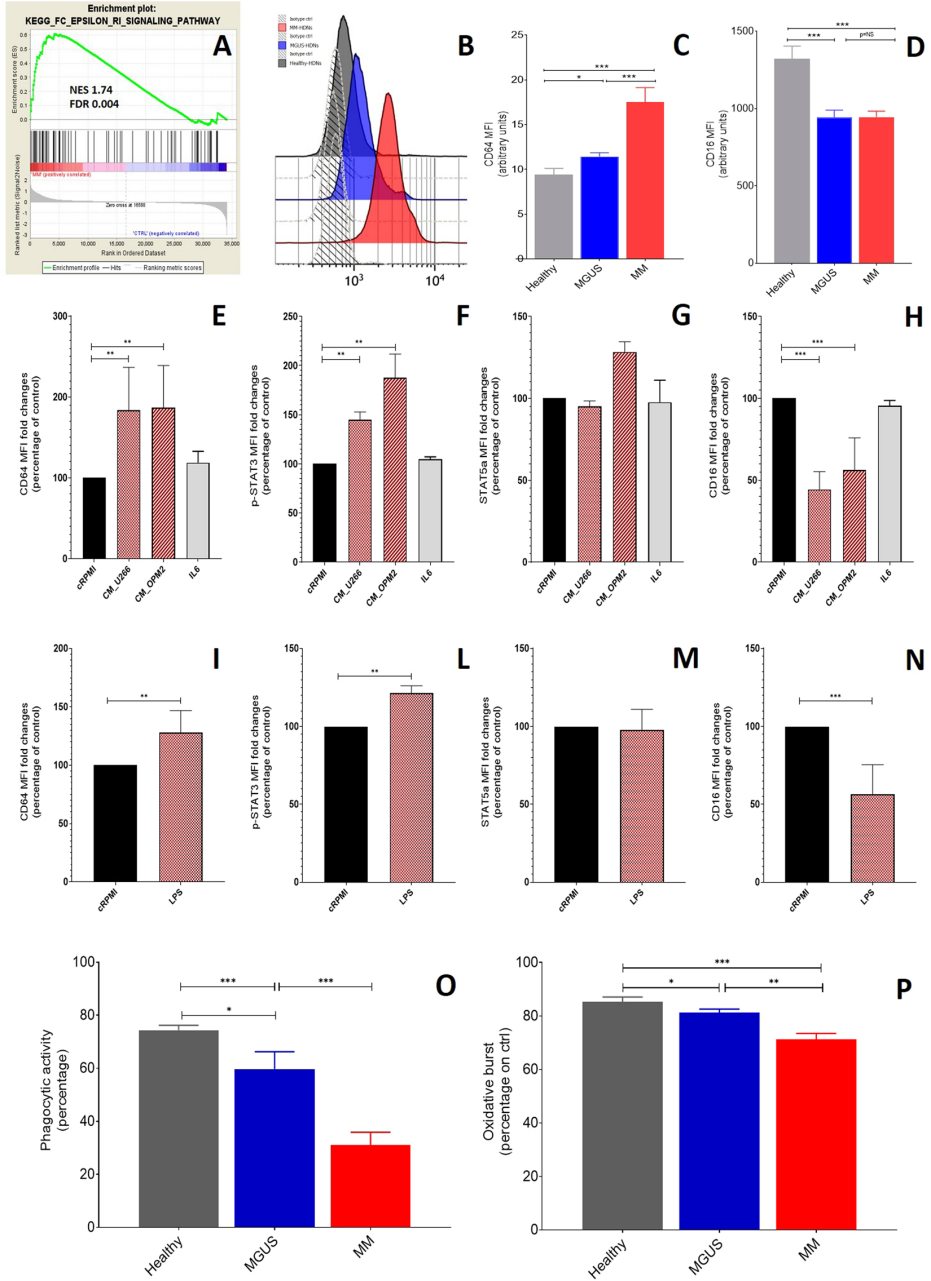
Neutrophil function in MM and MGUS high-density neutrophils is impaired as consequence of a MM-related soluble factors. (**A**) Gene set enrichment analysis of MM versus healthy HDNs identified an impairment in the FC-γ-receptor I (CD64) mediated phagocytosis. The green curves show the enrichment score and reflects the degree to which each gene (black vertical lines) is represented at the bottom of the ranked gene list. The heat map indicates the relative abundance (red to blue) of the genes specifically enriched in MM-HDNs as compared with the control cells. To validate the GEP findings, in an independent set of HDNs at steady state, as obtained from peripheral blood of MM, MGUS and healthy subjects, median intensity of fluorescence (MFI) of FC-γ-receptors CD64 (**B-C**) and CD16 (**D**) was detected by flow cytometry. (**E-H**) After exposure of healthy HDNs to MM conditioned media obtained from two human myeloma cell lines U266 and OPM2 or 500 ng/mL IL6 for 24 hours, CD64, p-STAT3, STAT5a and CD16 were measured by flow cytometry. (**I-N**) In the same experiments, the response to LPS was also evaluated. For more robust statistical evaluation, MFI values were converted to a resolution metric, such as the RD defined as (Median_treatment_ − Median_control_)/(rSD_treatment_ + rSD_control_) to further perform t-test to compare results of different experiments and runs. Stars denote p-value (***p < 0.0001) using t-test. After exposure to E-coli bacteria opsonized with IgG and complement of pooled sera, percentage of phagocytic activity (**D**) and oxidative burst (**E**) were detected by flow cytometry in controlled conditions in healthy (grey bars), MGUS (blue bars) and MM (red bars) HDNs. Stars denote p-value (*p < 0.005, **p < 0.001, ***p < 0.0001) using t-test for direct-pairwise comparison or ANOVA among three groups.

Since the GEP findings suggested that FcγRI-phagocytosis was impaired in MM-HDNs (Fig. 3A), we looked at the expression of Fc-gamma receptors: the inducible FcγRI, also known as CD64, and the constitutive FcγRIIIa, also known as CD16, both regulated by the amount of extracellular IL10 and IFN-γ^40,41^.

In an independent series, including 5 healthy, 15 MGUS and 15 MM subjects, we found that the amount of CD64 was higher in MM than in MGUS- (17.5 ± 1.6 versus 11.4 ± 0.4, p = 0.001) or healthy HDNs (MFI, 17.5 ± 1.6 versus 9.4 ± 0.7 a.u., p = 0.004, ANOVA test, Fig. 3C). Conversely, CD16 was lower in both MM- and MGUS-than in healthy HDNs (respectively MFI, 945 ± 38 versus 1323 ± 82 a.u., p < 0.0001, Fig. 3D), without any significant difference of CD16 expression between MGUS and MM-HDNs.

Exposure to sera obtained from peripheral blood of healthy (N = 3) or MM (N = 5) subjects reduced CD16 expression on surface of healthy HDNs (p = 0.003, ANOVA test), without affecting CD64, suggesting that MM-related soluble factors involved in the regulation of CD64 and CD16 expression are different (Supplementary Fig. 2A,B).

To assess the relationship between MM expansion and FcγRs modulation on HDNs, we assessed by flow-cytometry the CD64 and CD16 surface expression in healthy HDNs exposed for 24 hours to MM-conditioned media (obtained from two MM cell lines, U266 and OPM2) or IL-6, one of the most relevant cytokine in MM^44^. We found that MM conditioned medium, but not of IL-6, could increase CD64 amount by 24 hours (Fig. 3E), associated to increased amount of p-STAT3 (Fig. 3F), as expected^41^, but not of STAT5a (Fig. 3G). Furthermore, the pan-JAK2 inhibitor ruxolitinib, chosen to hamper the signaling downstream cytokine type II receptors IFNGR1/2, did not affect CD64 neither p-STAT3 or STAT5a expression (data not shown). Similarly, only the exposure to conditioned media and not IL6 could reduce CD16 expression on surface of normal HDNs (Fig. 3H).

Thus, we looked at soluble factors not related to plasma cells growth that could trigger STATs activation without affecting JAK1/2 downstream signaling. Since the cross-talk between FcγRI and bacterial component recognizing Toll-like receptors (TLRs) in human myeloid cells are critically involved in counteracting bacterial infections^45^, we triggered TLR2 exposing both healthy and MM-HDNs to LPS to appreciate the CD64 and CD16 at different time-points (Supplementary Fig. 2).

We found that in a time-course up to 24-hours, LPS treatment doubled CD64 and halved CD16 amount, in both healthy and MM-HDNs, within the first 6 hours (Supplementary Fig. 2C,D), in line to previously reported response to LPS via p-STAT3 in macrophages^46^. Indeed, at 24 hours, LPS increased about 20% the amount of baseline p-STAT3 (p = 0.007), without affecting STAT5a (Fig. 3I–N).

Based on these findings, we hypothesized that neutrophils in MM and MGUS are chronically activated as primed^45^ by gram-negative bacteria. Thus, we quantitative determined *in vitro* the percentage of neutrophils which had ingested *E.coli* bacteria opsonized with IgG and complement of pooled sera in controlled conditions. Surprisingly, we found that the percentage of phagocytic activity was lower in MM- and MGUS- than healthy HDNs (respectively, 30.9 ± 4.9% versus 74.4 ± 1.8 versus 73.6 ± 3.2%, ANOVA p = 0.001, Fig. 3O). In the same experiments, oxidative burst was lower in MM and MGUS- than healthy HDNs (respectively, 71.2 ± 2.3% versus 85.4 ± 1.7 versus 89.6 ± 1.2%, ANOVA p = 0.001, Fig. 3P).

### Arg-1, target of STAT-3, STAT-5 and STAT-6, is increased in both MGUS and MM-HDNs

We found ARG1 gene upregulation among the up-regulated genes in MM-HDNs compared to healthy HDNs. Since our previous work showed that ARG1, a transcriptional target of STAT-3^47,48^, STAT-5^49^ and STAT-6^50,51^, is increased in granulocytic-like myeloid derived suppressor cells in MM^28,38,52^, associated to inferior outcome after bortezomib treatment^28^, we explored its expression in both MGUS- and MM-HDNs.

**Figure 4:**
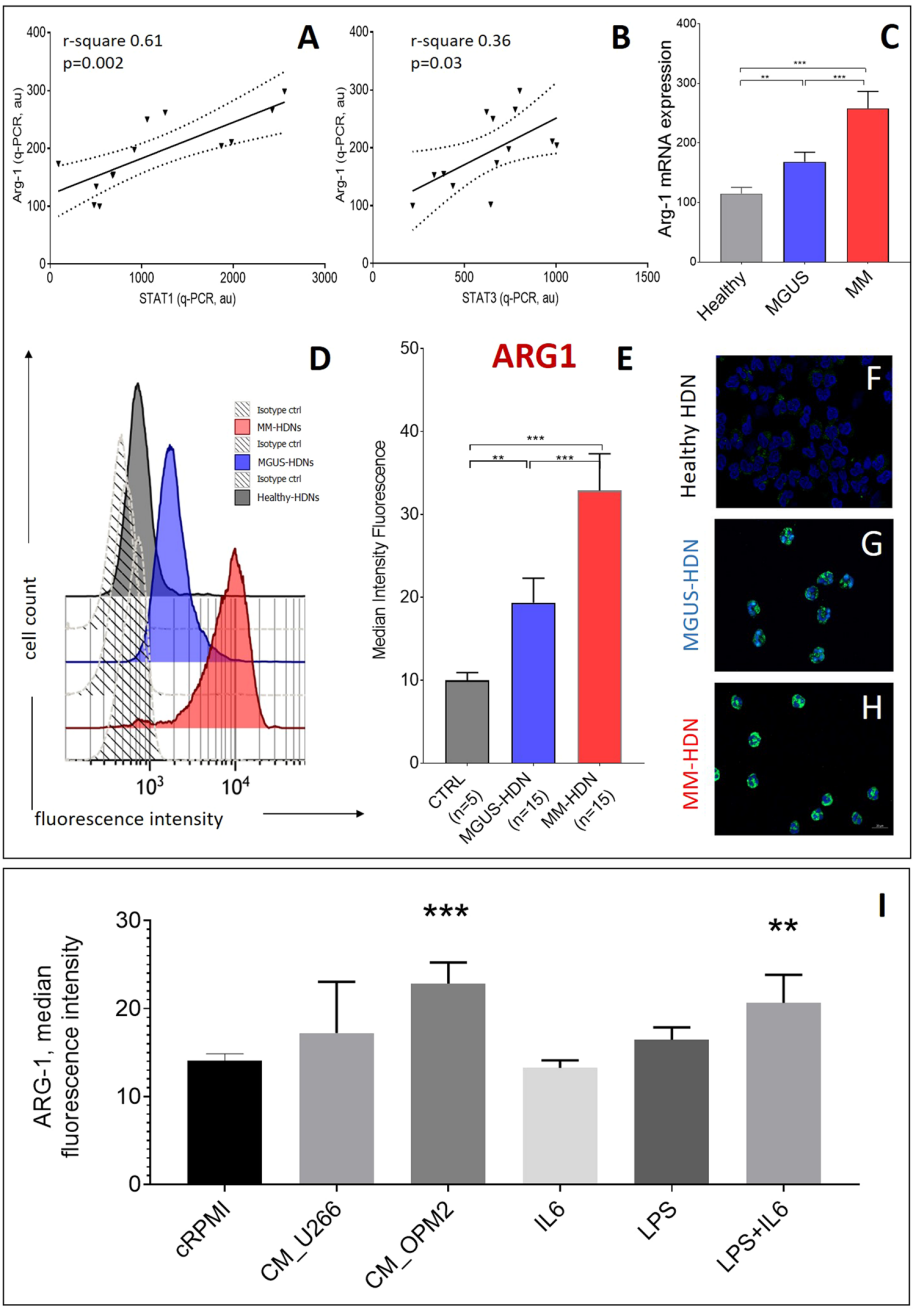
Arginase-1, target of activated STAT3, is increased in MM and MGUS high-density neutrophils. The association between the quantity of ARG1 transcript in MM and MGUS high-density neutrophils with STAT1 (**A**) and STAT3 (**B**) transcripts is shown. Dot-lines represent interval of confidence. (**C**) Arginase expression in healthy, MGUS and MM high-density neutrophils, as detected by qRT-PCR is shown; the differences were evaluated according to ANOVA test. In an independent set of HDNs at steady state, as obtained from peripheral blood of MM, MGUS and healthy subjects, median intensity of fluorescence (MFI) of ARG1 was detected by flow cytometry(**D-E**). (**F-H**) ARG1 immunofluorescence staining in HDN isolated by immune-magnetic-based positive selection after density gradient sedimentation from healthy, MGUS and MM subjects. ARG-1 localized in cytosol, in grains larger in MM-HDN than in controls. (**I**) After exposure of healthy HDNs to MM conditioned media obtained from two human myeloma cell lines U266 and OPM2 or 20 ng/mL IL6 or 100 ng/mL LPS for 24 hours, ARG1 was measured by flow cytometry. For more robust statistical evaluation, MFI values were converted to a resolution metric, such as the RD defined as (Median_treatment_-Median_control_)/(rSD_treatment_ + rSD_control_) to further perform t-test to compare results of different experiments and runs. Stars denote p-value (***p < 0.0001) using t-test.

The expression of ARG1 was positively associated to the increased amount of STAT-1 (r-square 0.61, p = 0.002, Fig. 4A) and STAT-3 (r-square 0.36, p = 0.03, Fig. 4B) transcripts, suggesting that it could be regulated downstream to the triggering of type II cytokine receptors. In an independent cohort of 5 healthy, 15 MGUS and 15 newly-diagnosed MM patients, ARG1 was progressively increased at both mRNA (ANOVA test, p = 0.004, Fig. 4C) and protein level, as detected by flow cytometry (Fig. 4D,E) and immunofluorescence (Fig. 4F–H).

Treatment for 24 hours with myeloma conditioned media obtained from OPM2 but not U266 HMCLs induced ARG1 in healthy HDNs (Fig. 4I), while nor IL6 neither LPS did not induce any change in the amount of intracellular ARG1. However, the combined exposure to LPS and IL6 was effective to overexpress ARG, as detected by flow cytometry (Fig. 4I).

### Arg-1 confers both MGUS and MM-HDNs immunesuppressive properties

**Figure 5:**
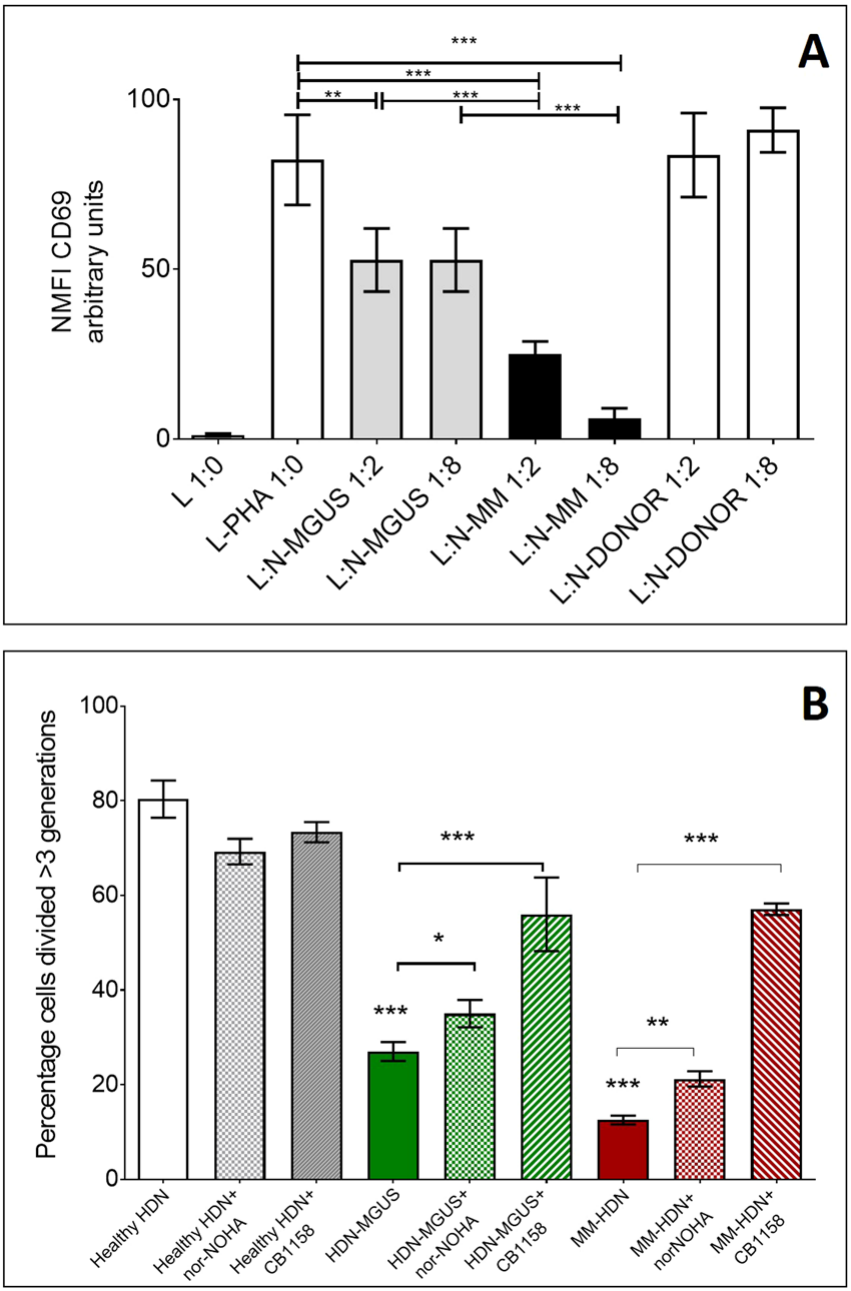
Arginase-1 confers both MGUS and MM high-density neutrophils immune suppressive properties. CD3 + T-cells obtained from healthy donors were activated using PHA and co-cultured with purified HDN freshly isolated from MGUS/MM patients or healthy donors, matched for sex and age, at increasing concentration (L:N ratio 1:2, 1:8). After 24 hours T-cells were examined for the early activation marker CD69. Bars represent the mean fluorescence intensity ± standard deviation of eight independent experiments, based on cells obtained from three different healthy donors, 6 MGUS and 6 MM/HDN (**A**). In an independent series of experiments, T-cells were labelled with CFSE and activated with PHA. After 3 hours, MGUS-HDN were added at the ratio L:HDN_*mgus*_ 1:4; alternatively, MM-HDN were added at the ratio L:HDN_*mm*_ 2:1 and cultured for 72 hours to measure proliferation. In the last 24 hours Arg-1 inhibitors nor-NOHA or CB1158 were added. Histograms show the percentage of proliferation of CFSE-labelled T-cells in presence of HDN and Arg-1 inhibitors (**B**). Stars denote p-value (***p < 0.001, **p < 0.05) using ANOVA test with post-hoc analysis. Abbreviations: L: lymphocyte, HDN: high-density neutrophils, PHA: phytohemagglutinin.

HDNs isolated from MGUS or MM patients were cultured with T-lymphocytes obtained from healthy volunteers (Fig. 5A). After 72 h from mitogen stimulation (PHA), we observed that MM-HDN reduced T-cell activation at both tested 1:2 and 1:8 ratios (Fig. 5A, Supplementary Fig. 3) and proliferation at both tested 1:2 (data not shown) and 1:8 ratios (14.3 ± 0.6%, p < 0.0001, Fig. 5B). In presence of MGUS-HDNs, the reduction of T-cell activation was similar at 1:2 and 1:8 ratio, while defective T-cell proliferation was evident only at the 1:8 ratio (25.4 ± 4.3%, p = 0.002).

In both MGUS and MM-HDNs, the immune suppressive activity was reverted by treatment with two ARG-1 inhibitors, nor-NOHA or CB-1158. The revert of immune-suppression was more efficient with CB-1158 than nor-NOHA for the concomitant overexpression of MOSC1, a component of an N-hydroxylated prodrug-converting complex, able to reduce Nω-hydroxy-L-arginine (NOHA) into L-arginine, providing a collateral escape from ARG-1 inhibition (Supplementary Fig. 4).

### FcγRI-CD64 in MM-HDNs is associated to clinical response to bortezomib and thalidomide

**Figure 6:**
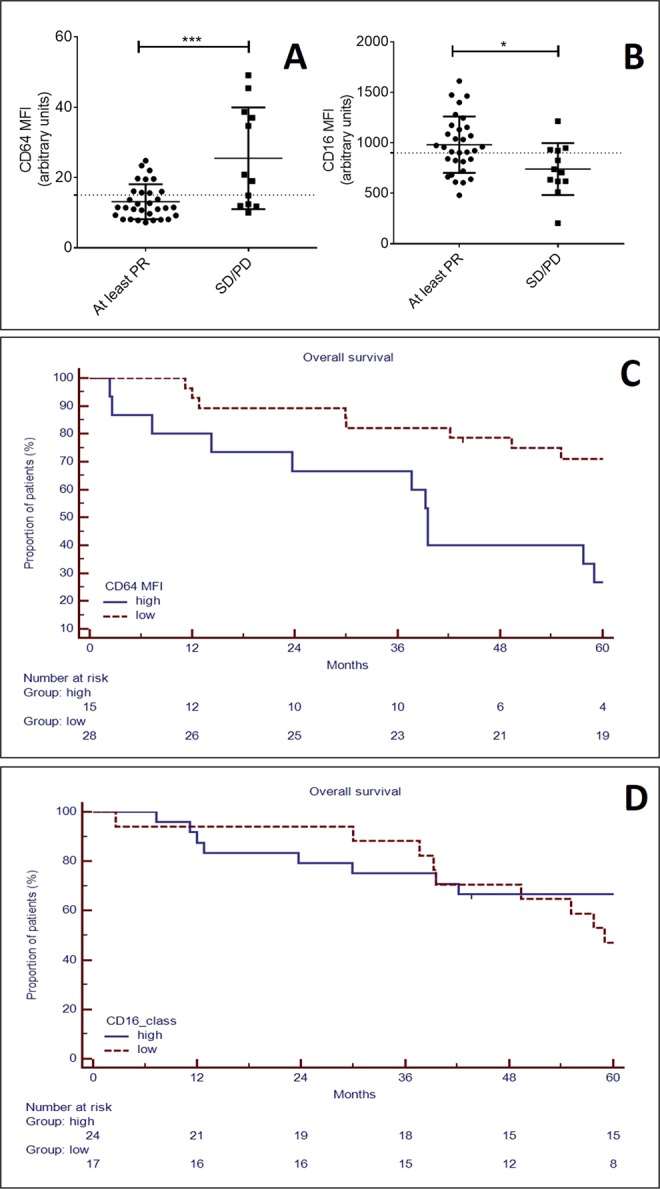
Intensity of CD64 on high-density neutrophils can predict overall survival in multiple myeloma patients. In circulating neutrophils, identified as CD45^+^CD11b^+^CD15^+^CD14^−^ cells, obtained from peripheral blood of healthy, MGUS and MM patients we measured the mean of fluorescence intensity of surface expression of CD64 (**A**) and CD16 (**B**). Stars denote p-value (***p < 0.001, *p < 0.05) using t-test. Based on the reduction of monoclonal component in serum/urine patients and consequently response after 4 cycles of induction treatment, patients were distinguished in two groups: those who obtained at least a partial response (equivalent to reduction of at least 50% of monoclonal component from baseline) or not (identified as stable or progressive disease). Overall survival was defined as time from the date of initial diagnosis to the date of death for any reason or last follow-up in two groups of patients distinguished based on CD64 (**C**) or CD16 (**D**) MFI, using as threshold the mean value plus 2 standard deviation found in healthy subjects.

Since surface expression of FcγRs has been successfully tested as biomarker of proven bacterial infection, with clinical implications, we explored the role of CD64 and CD16 expression on HDN in the MM outcome. Between January 2012 and April 2013, 43 consecutive newly diagnosed MM patients (Supplementary Table 4), candidates to autologous stem cell transplantation, received first-line treatment based on bortezomib, thalidomide and dexamethasone (VTD). We correlated CD64 and CD16 expression on HDN at diagnosis and response after first four induction cycles: 31 patients achieved at least a partial response (6 complete remission, CR; 17 very good partial response, VGPR and 8 partial remission, PR, according to IMWG 2011 response criteria^53^, while 12 failed to obtain any response (6 stable disease, SD and 6 progressive disease, PD). Patients who obtained at least a partial response had lower CD64 MFI (13.1 ± 0.9 versus 25.5 ± 4.2, p = 0.0001) and higher CD16 MFI (980.6 ± 50.1 versus 739.5 ± 74.1, p = 0.01, Fig. 6A,B). Using as threshold the mean value plus 2 standard deviations in healthy subjects, we found that high CD64 could identify at diagnosis patients with inferior median overall survival (39.5 versus 86.7 months, p = 0.04, Fig. 6C), while CD16 could not (Fig. 6D). In univariate analysis high CD64 MFI, high LDH, low hemoglobin levels and stage 3 ISS at baseline were associated to lower 5-years overall survival (Table 2).

**Table 2:** Univariate and multivariate analysis of OS in 43 patients treated up-front with VTD.

n			Univariate analysis		Multivariate analysis	
			OS @ 36 months	p-value	HR (95% CI)	p-value
Gender	male	30	63.3	0.12		
	female	13	43.3			
ISS	I-II	24	75.0	***0.01***	1.5 (0.5–4.3)	0.40
	III	19	31.6			
FISH risk	standard	38	60.0	0.74		
	high	5	52.2			
Hb < 10 g/dL	no	11	81.8	0.05		
	yes	32	46.2			
LDH > 2 UPN	no	28	89.7	***0.0004***	1.7 (0.7–3.9)	0.21
	yes	15	39.5			
Histotype	IgG	36	75.0	0.82		
	IgA	4	52.5			
ClCr	<30 ml/min	8	53.9	0.78		
	>30 ml/min	35	62.5			
CD64 MFI on CD15 + cells	normal	28	71.1	***0.004***	***2.3 (0.9–5.8)***	***0.07***
	high	15	26.7			
CD16 MFI on CD15 + cells	normal	24	66.7	0.46		
	low	17	41.2			

## Discussion

In this work, we investigated immune-phenotype and function of mature, high-density neutrophils circulating in peripheral blood of MM and MGUS patients, aiming that HDNs could have a role in the complex pathogenesis of myeloma development and mirror the interplay between the tumor growth in the bone marrow and the myeloid development. To exclude the contamination with eosinophils or monocytes, we worked with highly-purified freshly-isolated HDNs^38,54^.

The transcriptional program of both MGUS- and MM-HDNs suggests that, in comparison with HDN from normal subjects, they are chronically activated, up-regulate CD64 and downregulate CD16, have a defective phagocytic activity and they are immune suppressive due to ARG1 over-expression. The role of the immune-suppressive myeloid compartment in favoring progression of disease in MM has been demonstrated both in experimental^35,36,55,56^ and clinical models^57–61^, however this is the first study reporting changes in human HDNs during an hematological malignancy, developed in the bone marrow, where the tumor cells share the same site with myeloid progenitors. Our findings clearly indicate that the combination of phenotypic changes (increased CD64 and Arg-1) and function (reduced phagocytosis and increased immune-suppression) occurring in MM-HDN overlap with those described for G-MDSC in MM, in line with recent reports in the field^62^, while HDN in healthy subjects do not have immunosuppressive features. Key MDSC genes and canonical signaling pathways are activated along tumor progression, as recently demonstrated in murine cancer models, where a consensus set of 817 genes, involved in myeloid cell recruitment and angiogenesis, was identified^63^.

Despite the transcriptome of MGUS and MM HDNs is very different, with less than 10% of overlapping alterations compared to healthy subjects, in both we found a gene-signature converging on STATs activation, as consequence of an early or chronic response to soluble factors. In the attempt to recapitulate *in vitro* changes seen *in vivo*, we found that soluble factors not related to MM expansion, like LPS, part of gram-negative bacterial membranes, could affect HDN immune phenotype.

Some reports suggest that circulating (complete or incomplete) IgG proteins could trigger neutrophil activation^64^ but in our series neutrophilic dysfunction was not associated to any specific isotype (data not shown).

In human and mouse models, MM-derived exosomes can hamper myeloid maturation, conferring immature myeloid cells potent immune suppressive activity, through activation of STAT3, leading to increased immunosuppression which favors MM progression^65,66^. Our previous work showed that immune-modulatory factors released by MM-mesenchymal stem cells are able to induce G-MDSC *in vitro* from healthy peripheral blood mononuclear cells, supporting an evolving concept regarding the contribution of MM microenvironment to tumor development and progression through immune-editing^52,67^. While in solid tumors HDN could reflect the composition of immune infiltrate in tumor microenvironment without exerting immune-suppressive activity, HDN in MGUS and MM patients are immune-suppressive and share immune-suppressive features with G-MDSC, associated to increased CD64 and Arg-1, reduced phagocytosis and CD16^25,34,60^. In MM-murine model HDN and G-MDSC exert the same chemo-protective role without sharing the immune-suppressive property^35^, but novel insights in the field suggest that the cancer-related myelopoiesis could be different in mouse and human setting. Moreover, we found that in MGUS- and MM-HDNs the enzyme MOSC1 was higher than controls, contributing stem and thus might represent another physiological regulatory detox mechanism of neutrophils in response to inflamed environment^68^. The overexpression of MOSC1 could explain the differences in the recovery from arginase inhibition that we have reported in the co-cultures of HDNs in presence of T-cells and arginase inhibitors nor-NOHA and CB1158.

**Figure 7:**
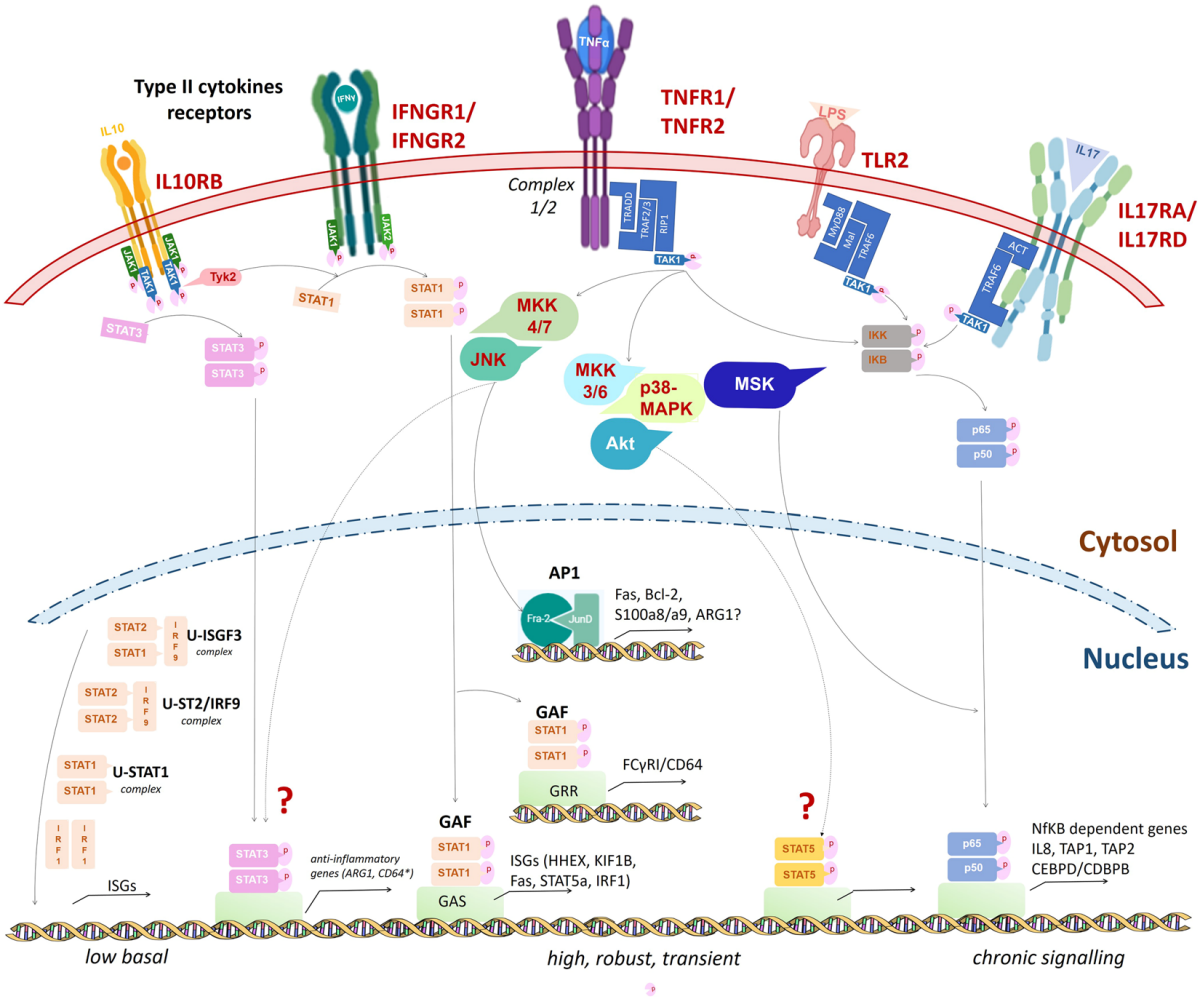
Pathways transcriptionally dysregulated in MGUS- and MM- HDNs. Our work showed that MM-HDNs had increased expression of IL10RB, IFNGR1/2, TNFR1/2, TLR2, IL17RA/D. While IFN-gamma can activate IFNGR1/2 and IL10 can bind IL10RB in response to unresolved chronic inflammation, to activate STAT1 and STAT3 and promote their nuclear translocation, LPS triggers TLR2, through an adaptor complex which recruits TRAF6 to activate the TAK1 kinase complex can then activate the IKK complex leading to NFkB activation. The increased expression of TNFR1/B and component of their adaptor complex ½ can recruit several transcription factors to amplify the cascade and warrant a robust response. The genes target CD64 and ARG1 are under the transcriptional control of STAT1 and STAT3, as previously reported for other professional phagocytes. The lack of IL17RC excludes that the IL17R complex could be active, but the ligand of the overexpressed IL17RD is still unknown.

The regulation of CD64 expression in phagocytes is under control of IFN-γ that can induce the increased phosphorylation of STAT3 to bind the GRR motif in the gene promotor (Fig. 7)^40,41^. Despite in monocytes the CD64 overexpression is driven by both IL10 and IFN-γ and we have found the overexpression of ILR10B and its downstream effectors, we have not tested IL10 *in vitro* to recapitulate the MM-HDN phenotype since it is known that normal neutrophils do not upregulate CD64 via IL10, but only via IFN-γ^13,40,41,69–71^. There is an emerging interest in identifying the molecular machinery involved in the IFN response in the MM microenvironment, since several cellular types show an IFNAR1 related signature in response to type 1 interferon secretion by myeloma cells^72^, while type 3 interferon is reduced^73^ due to anergic NK and T-cells^61^ also in the asymptomatic MGUS phase^74^. Further effort in our lab is currently focused to identify the source of extracellular IFN-γ in the paracrine loop sustaining MM expansion, and we have evidence of out-of-lineage, low-rate synthesis of IFN-γ in MM-HDNs^38^.

We found that both MGUS and MM-HDNs have a reduced phagocytic activity, that could determine an increased susceptibility to infection in both settings^6,8,11^ as well as an ineffective anti-tumor response^75,76^. The defective phagocytosis could limit antigen presentation by professional APC^77^ or play a role in the neutrophil-mediated antigen presentation to CD4^+^T-cells, contributing to immune suppression. It has been reported that bacterial infections contribute to MM progression from an asymptomatic phase, and that TLR2 polymorphisms are associated to increased susceptibility to certain bacterial infections (*S. Aureus, S. pneumoniae, L. monocytogenes, M. tubercolosis*)^78^. For example, in the chronic tuberculosis infection^79^, neutrophils upregulate TLR2 and CD64, but have reduced phagocytosis. We hypothesize that in response to signaling triggered by soluble factors, such as IL8 and IL17, which are increased in MM microenvironment, the HDN function can change. The reduced phagocytosis in MM neutrophils has been described also in patients with low tumor burden, at 20 days after autologous stem cell transplantation, suggesting that factors not produced by MM cells can be implied in the neutrophil impairment^80^.

Despite we found an increase of several components of the IL17 signaling (IL17RA, TRAF6, TYK2) and its downstream effectors CEBPD and CEBPB in both MM and MGUS HDNs, we did not test *in vitro* the effect of IL17 on neutrophils function, because of lack of ILR17C, required for the full receptor assembly to work properly^81,82^. We also found that IL17RD is higher in MM- than MGUS-HDNs, but its ligand is still unknown^82^; it is questionable if it could be involved in the STAT3 activation exerted by soluble mediators released by the MM- conditioned media. On the other hand, the paracrine effects of IL17 can be due to the gram-negative microbiome composition, able to affect the evolution from MGUS to MM, as recently shown in Vk*MYC mice^83^. In the future, it will be worth to investigate the genetic and environmental predisposition to bacterial infections to personalize the immune therapy in MM.

Our results were not conclusive to determine if CD64 and CD16 could reflect or not the contribution of intrinsic and extrinsic signals related to neoplastic plasma cells expansion, even if several groups suggested a strong connection between innate and adaptive immune responses via TLRs signaling. Indeed, the main targets of LPS (used in our experiments *in vitro*) are TLR2 and TLR4, upregulated in both neoplastic plasma cells^84^ and MM microenvironment supportive cells^85^, and we found an increased expression of TLR2 and its downstream gene targets, like IL8.

Our work suggests a contribution of HDNs to hamper the T-cell function in MM via ARG-1^59,86–88^, that could be a novel target to improve adoptive T-cell therapy, especially in early phase of disease onset. There is an emerging interest in addressing biomarkers of immune function, since patients with normal immunological function, like recovery of γδ T cells that respond to infections and tumor antigens as components of innate and adaptive immunity^59,86,87^, have better outcome^89^.

Therefore, MM-HDN fully mirror myeloid-driven immunosuppressive function and can be adopted in the future in reproducible assays to globally estimate the role of the host aberrant myelopoiesis in promoting progression of MM and to evaluate immune recovery in patients after treatment.

In this perspective, quantification of CD64 on HDNs could be helpful. In sepsis, the quantitative expression of CD64 in neutrophils could discriminate between sepsis and non-septic systemic inflammatory response syndrome, with translational relevance^90–92^. Based on several studies in the field, the amount of CD64 expression during the first 24 hours of suspected clinical infection can allow clinicians to discontinue unnecessary antimicrobial treatments with no need to wait for confirmation by microbiological testing^90–92^. We disclosed that patients carrying high CD64 on neutrophils have inferior outcome and recovering their immune function could be a novel therapy goal^64^. There is an emerging interest in addressing biomarkers of immune function, since patients with normal immunological function, like recovery of γδ T cells that respond to infections and tumor antigens as components of innate and adaptive immunity^59,86,87^, have better outcome^89^.

Taken together, our findings confirm the impairment of the immune function since the early asymptomatic phase of MGUS, as previously showed in murine models and human disease for other cell types^35^. HDNs act as hub of cancer-related and bacteria-related crosstalk, due to upregulation of several receptors that fine tune external dampening signals conveying to chronic STATs activation. These alterations could have a role in progression of disease and in early events occurring in the microenvironment dysregulation, since MGUS-HDN show a phenotype and functional pattern of partial dysfunction and limited immunosuppressive activity. In clinical progression through MM, neutrophils may play a role in supporting both the increased susceptibility to infections and the immunological suppression that leads to tumor progression. The association between defects of the innate immunity and increased cancer susceptibility is emerging. For examples, single nucleotide polymorphisms of TLR2 and increased expression of CD64 are more frequent in Afro-American descents and are associated to increased risk of prostate cancer^93,94^. Further studies will be needed to address if infection susceptibility can affect the trajectory of myeloma progression from MGUS.

## Methods

### Patients and controls

Between January 2013 and December 2014, 60 newly diagnosed multiple myeloma (MM) and 30 monoclonal gammopathy of uncertain significance (MGUS) patients have been included in this study (Supplementary Table 1). Patients were free from immune-mediated diseases and acute or chronic viral infections to avoid any interference on immune-regulatory mechanisms. All MM patients had measurable disease. All MGUS patients had a stable chronic disease with at least 2 years of follow up. Thirty healthy subjects (age > 45 years) were recruited in the study as controls.

None of the recruited patients was receiving medical treatments that could have an impact on their immune condition. All subjects involved in the study provided their written informed consent according to the Declaration of Helsinki, as approved by the Ethical Local Committee Board *(Comitato Etico Catania 1, #19351, 04.12.2009*, https://www.policlinicovittorioemanuele.it/comitato-etico-catania-1*)*.

### Isolation of Neutrophils and Lymphocytes

Whole blood (40 ml) was collected from healthy volunteers, MGUS and MM patients in vacutainer tubes containing the anticoagulant, potassium EDTA and diluted 1:1 with dextran 3% for two hours to obtain plasma enriched of white cells. Peripheral blood mononuclear cells (PBMC) were then isolated by the standard method of density gradient centrifugation using Ficoll-Paque (Pharmacia LKB Biotechnology). The resulting interphase layer from the gradient was diluted and washed twice with Dulbecco’s phosphate buffered saline (PBS, Celbio) to obtain PBMC from the top and neutrophils from the bottom. T-lymphocytes were isolated using T-cells enrichment columns (R&D Systems). Purity of T-lymphocytes (>95%) was assessed using flow cytometry.

To isolate neutrophils, the pellet obtained after centrifugation of PB on Ficoll, containing erythrocytes and high-density neutrophils (HDN), was subjected to hypotonic lysis (155 mM NH4 Cl, 10 mM KHCO3, 0.1 mM EDTA, pH 7.4) for 15 minutes on ice. After washing, cells were further immune-magnetically sorted using the EasySep human neutrophil Isolation kit (StemCell Technology, cat #17957)^54^. HDN purity, maturity and viability were checked by morphology, as previously described^38^ and flow cytometry as CD45^+^CD11b^+^CD15^+^CD14^-^cells (Supplementary Fig. 5). HDNs with purity and viability of more than 95% were used for further assays, as previously described^38^.

### Gene expression profile of HDNs

RNA concentration was determined with a Nanodrop (Nano-Drop, Wilmington, Delaware, USA) spectrophotometer and its quality was assessed with an Agilent 2100 Bioanalyzer (Agilent Technologies, Milano, Italy).

For each sample, 300 ng of total RNA was reverse transcribed and used for synthesis of cDNA and biotinylated cRNA according to the Illumina TotalPrep RNA amplification kit protocol (Ambion, Austin, TX; category number IL1791). Hybridization of 750 ng of cRNA on Illumina HumanHT12 v4.0 Expression BeadChip array (Illumina Inc., San Diego, CA, USA), staining and scanning were performed according to the standard protocol supplied by Illumina. The analysis was performed in duplicate for each sample. BeadChip was dried and scanned with an Illumina HiScanSQ system (Illumina Inc.).

For data analysis, we used two approaches. First, the intensity files were loaded into the Illumina Genome Studio software for quality control and gene expression analysis. Quantile normalization algorithm was applied on the data set to correct systematic errors, values below a detection score of 0.05 were filtered out and missing values were imputed. Differently expressed genes (DEGs) were selected with differential score (DiffScore) cutoff set at ± 13 (p > 0.05). The DEGs list (composed by 894 genes, 551 up- and 343 down-regulated) was used to evaluate the gene ontology performing an enrichment analysis with DAVID Bioinformatics (http://david.abcc.ncifcrf.gov/home.jsp^95^)- in terms of Biological Processes, Cellular Component and Molecular Function. The degree of enrichment was statistically evaluated to determine whether an observed level of annotation for a group of genes was significant. The statistical significance of the gene ontology was computed using a modified Fisher Exact test (EASE score), the resulting P-values were corrected using the Benjamini and Yekutieli FDR methods. Enrichment analysis was calculated to rank overall importance of annotation term groups. It is the geometric mean of all the enrichment P-values (EASE scores) of each annotation term in the group.

Second, Gene Set Enrichment Analysis (GSEA) was performed using the publicly available desktop application from the Broad Institute (http://www.broad.mit.edu/gsea/software/software_index.html). The gene sets database used was that of functional sets, s2.symbols.gmt. P values were calculated by permuting the genes 1,000 times. The classic enrichment statistic was selected, as described above.

To understand either common or unique pathways and protein networks we performed a final analysis with the recently developed Metascape suite^39^. Data are available at http://www.ebi.ac.uk/arrayexpress/help/FAQ.html#cite, ArrayExpress accession E-MTAB-6105.

### Expression of immunosuppression-related genes

Total RNA was extracted from neutrophils using Trizol reagent and quantified using UV spectrophotometer. For real-time PCR analysis of mRNA expression, 1.0 μg of total RNA (in 20 μl reaction volume) was reverse transcribed using reverse transcriptase (Roche Diagnostic Corp., Indianapolis, IN, USA) and oligo-dT primers in a standard reaction. The resultant cDNA was then used as the template for PCR amplification. The quantitative real-time polymerase chain reaction was performed by use of a LightCycler (Roche), with primers designed specifically for the transcripts of ARG1 (Fw: ACAGTTTGGCAATTGGAAGCA Rv: CACCCAGATGACTCCAAGATCAG), MOSC1 (Fw: GCTTCCTGAAGTCACAGCCCTA Rv: CAAGAATGGGCTGGTGTCTGAG), STAT3 (Fw: GAGAAGGACATCAGCGGTAAG Rv: AGTGGA GACACCAGGATATTG), STAT1 (Fw: GGCAAAGAGTGATCAGAAACAA Rv:GTTCAGTGACATTCAGCAACTC) according to the gene manufacturer’s recommended protocol (Applied Biosystem). Each reaction was run in triplicate. Samples were quantified accordingly (LightCycler analysis software, version 3.5) using the housekeeping gene GAPDH (Fw: TCCTGTTCGACAGTCAGCCGCA, Rv: GCGCCCAATACGACCAAATCCGT) as standard.

### Immunephenotype of HDNs

One hundred thousand HDNs were separated using a combination of physical and immune-magnetically based methods as described above were stained with the following antibodies from Beckman Coulter: CD15-FITC (clone 80H5), CD11b-PE (clone bear-1), CD14-PC7 (clone RMO52) and CD45-ECD (clone J33).

In selected experiments HDNs were identified by forward and 90° light scatter parameters, CD11b and CD15 expression and stained with the following antibodies from Beckman Coulter: CD11b PC5 (clone bear1), CD15-FITC (clone80H5), CD64 ECD (clone 22); CD16 ECD (clone - 3G8); Biolegend: Arg-1-PE (clone, 14D2C43); and Mylteni: STAT3pS727 PE(clone REA324), STAT5a-PE (clone REA549), STAT6-pY641- FITC (clone REA 413), and respective isotypic controls.

Samples were then washed in PBS and mean fluorescence intensity (MFI) corrected for values of nonspecific binding was acquired by a Navios flow-cytometer.

### Quantification of phagocytosis and oxidative burst

Phagocytic and oxidative burst activities were detected from heparin blood using the Phagotest kit (Opregen Pharma, Heidelberg, Germany) and Phagoburst kit (Glycotope Biotechnology GmbH, Heidelberg, Germany), following manufacturer’s instructions. Cells were gated through the scatter parameters (forward, FCS versus side scatter, SSC) and their green fluorescence histogram was analyzed by a Navios flow cytometer. The results were expressed as percentage of fluorescent cells in the population studied and calculated by subtracting the percentage of the negative control sample (<1%) from the positive sample.

### Evaluation *in vitro* of changed in surface markers of healthy HDNs after treatment with MM-related soluble factors

For sera collection obtained from MM (N = 8) and MGUS (N = 6) subjects, blood was centrifuged by 2 hours at 1600 × g for 10 minutes at room temperature and surnatant saved at –80 °C for maximum 2 months. OPM2 and U266 human myeloma cell lines (HMCLs) were kindly provided by prof. Tassone (Magna Grecia, University of Catanzaro), previously validated using sequencing and phenotypic characterization^96^, were plated 72 hours prior to collection of conditioned media, which was then filtered using a 0.2μm syringe filter, diluted to the appropriate concentration with complete RPMI.

HDNs, isolated from 8 healthy donors, were incubated with 5% CO_2_ at 37 °C at the concentration of 100 × 10^4^/ml with sera 20% obtained from 8 MGUS and 8 MM patients (matched for sex and age) or conditioned media to evaluate changes in surface expression of CD64 or CD16 after 24 hours. In another set of experiments, healthy HDNs (100 × 10^4^/ml) were grown in 12-wells tissue culture plates in RPMI1640 Gibco) supplemented with 10% FBS and 1% penicillin/streptomycin and treated for 24 hours with recombinant human IL-6 (Biolegend, 715104), LPS (Cell Signaling Technology, #14011) or both, at final concentrations respectively of 20 ng/ml and 100 ng/ml. At the end of treatments, cells were collected and stained as previously described to detect CD64, CD16, Arg-1, STAT3pS727 and STAT5a, then washed with PBS, centrifuged for 5 min and resuspended in 300 μL of PBS for further flow cytometry analysis.

For more robust statistical evaluation, MFI values were converted to a resolution metric, such as the RD defined as (Median_treatment_ − Median_control_)/(rSD_treatment_ + rSD_control_) to further perform t-test to compare results of different experiments and runs.

### Detection of Arg-1

For immunofluorescence analysis of Arg-1 in neutrophils, the sections were incubated overnight in a humid chamber at 4 °C with primary antibody anti-Arg-1 (anti-rabbit, Sigma-HPA003595). After a rinse in PBS for 10 min, the sections were incubated for 2 h at room temperature with fluorescein isothiocyanate (FITC) conjugated goat anti-rabbit IgG (Sigma). In order to identify five fields with the largest number of immunostained cells, a first observation of the tissue sections under a 20 × objective was carried out. Then using 40 × oil-immersion objective, the counting of the immune-positive cells was performed in each one of these fields. All observations were made with a Zeiss Axio Imager Z1 epifluorescence microscope (Carl Zeiss AG, Werk Göttingen, Germany), equipped with an AxioCam camera (Zeiss, Jena, Germany) for the acquisition of images.

### Evaluation of suppressive activity of HDNs against allogeneic T-cells

50 × 10^4^ allogenic T-lymphocytes isolated from 5 healthy donors were labeled with 1 µM of Carboxyfluoresceinsuccinimidyl ester (CFSE) (BD Pharmingen) at 37 °C for 20 min, then added to a 24-well tissue culture plate in the presence of phytohaemagglutinin (PHA-P, 5 mg/ml) (Sigma-Aldrich) to induce T cell proliferation and co-cultured with neutrophils (N) obtained from 5 MM, 5 MGUS or 5 healthy subjects (matched for sex and age) at ratio 1:2 and 1:8. The T-cell proliferation measured by CFSE dilution was evaluated by flow cytometry after 72 hours.

In addition, T-lymphocytes from 5 healthy donors were harvested, washed twice in staining buffer (PBS containing 0.2% BSA and 0.1% sodium azide) and added to a 96-well polypropylene plate at concentration 5 × 10^5^ cells/well. After stimulation with 5 mg/ml PHA they were co-cultured with neutrophils (N) obtained from 5 MM, 5 MGUS or 5 healthy subjects at ratio 1:2 and 1:8.

Then, at each time point (24, 48 and 72 hours), we evaluated the expression on T-lymphocytes of some activation markers such as HLA-DR, CD69 and CD71. PHA-stimulated T-cells were used as control.

For flow-cytometry analysis, 50 × 10^4^ cells/well were stained with the following monoclonal antibodies purchased from Beckman Coulter: HLA-DR- PC5 (Clone Immu-357), CD3 ECD (clone UCHT1), CD69 PE (clone TP1.55.3), CD71 FITC (clone YDJ1.2.2). Staining with respective isotype-matched control antibodies was also included for each condition to detect nonspecific background staining. Density of expression of activation markers (Mean Fluorescent Intensity, MFI) were obtained and represented.

### Statistical methods

Statistical analyses for subject data and functional analyses were performed using Prism GraphPad Software (La Jolla, CA, USA). Two-tailed Student’s t-tests and Fischer’s exact tests were used to compare continuous and categorical clinical variables, respectively. Mann-Whitney U test was utilized for non-parametric data. For each neutrophil function measurement (phagocytosis and oxidative burst, CD64 and CD16 expression), means and standard deviations, median and IQR for non-symmetric variables were calculated and compared among healthy, MGUS and MM subjects by use of a two-tailed Student’s t test with Bonferroni correction. ANOVA test was used to compare means of more than two groups. Descriptive statistics were generated for analysis of results and p-value under 0.05 was considered significant. Qualitative results were summarized in MFI (mean fluorescence intensity) and in percentages.

Using as threshold the mean value plus 2 standard deviation in healthy subjects of both CD64 and CD16 MFI we identified four groups, designated as “low-CD64”, “high-CD64”, “low-CD16” and “high-CD16”. These thresholds were then carried forward to predict overall survival.

Overall survival (OS) was defined as time from the date of initial diagnosis to the date of death for any reason or last follow-up. Follow-up times were described as medians by using the inverse Kaplan-Meier estimator [28]. Survival curves were obtained by using the Kaplan-Meier method and were compared with the log-rank test. The Cox proportional hazards model was used to calculate adjusted hazard ratios (HRs) and their 95% confidence intervals (CI). All statistical tests were two-sided, and the threshold for statistical significance was p = 0.05, performed using MedCalc for Windows, version 12.5 (MedCalc Software, Ostend, Belgium).

### Ethics declarations

FDR, AR and CC received honoraria from Celgene, Takeda and Amgen; GAP and FDR received honoraria from Novartis. All the other authors declare no competing interests.

## Supplementary information

Supplementary tables and figures.

## Data Availability

All data generated or analyzed during this study are included in this published article (and its supplementary files) and in public repository: http://www.ebi.ac.uk/arrayexpress/help/FAQ.html#cite, ArrayExpress accession number E-MTAB-6105.
