# Supplementary material for: High-density neutrophils in MGUS and multiple myeloma are dysfunctional and immune-suppressive due to increased STAT3 downstream signaling
Source: Sci Rep. 2020 Feb 6;10:1983. doi: 10.1038/s41598-020-58859-x (PMC7005058; doi:10.1038/s41598-020-58859-x)
Supplement: Supplementary file 1 — Supplementary tables and figures. [file 41598_2020_58859_MOESM1_ESM.docx]

**High-density neutrophils in MGUS and multiple myeloma are dysfunctional and immune-suppressive due to increased STAT3 downstream signaling**

A. Romano^1^*, N.L. Parrinello^2,3#^, V. Simeon^4#^, F. Puglisi^2^, P. La Cava^2^, C. Bellofiore^1,2^, C. Giallongo^2^, G. Camiolo^2^, F. D'Auria^4^, V. Grieco^4^, F. Larocca^4^, A. Barbato^2^, D. Cambria^2^, E. La Spina^5^, D. Tibullo^5^, G. A. Palumbo^2,3^, C. Conticello^2^, P. Musto^4^ and F. Di Raimondo^1,2*^

^#^*These authors are co-authors*

**Authors’ affiliations**

^1^ Department of Surgery and Medical Specialties, University of Catania, Catania, Italy

^2^ Division of Hematology, Azienda Ospedaliera Policlinico e Vittorio Emanuele di Catania, Italy

^3^ Dipartimento di Scienze mediche, chirurgiche e tecnologie avanzate, University of Catania, Italy

^4^ Laboratory of Pre-Clinical Research and Advanced Diagnostics, IRCCS-CROB, Rionero in Vulture (Pz). *VS current address: Department of Mental Health and Preventive Medicine, Medical Statistics Unit, University of Campania “Luigi Vanvitelli”, Naples, Italy;

^5^ Biometec, Dipartimento di Scienze Biomediche e Biotecnologiche, University of Catania, Italy

**Running Title**: Neutrophil impairment in MGUS and MM

**Key Words**: MGUS; multiple myeloma, neutrophils, phagocytosis, arginase

**Corresponding Author**: Francesco Di Raimondo

**Address:** Department of Surgery and Medical Specialties, University of Catania, Catania, Italy

Via Santa Sofia, 78

95124, Catania, Italy

**e-mail address**: diraimon@unict.it

**phone number**: +39 095 378 1956

**fax number**: +39 095 378 1978

**Scientific Category**: Original Research Paper

**Figures:** 6 main figures + 5 supplementary figures

**Tables** 2+4 supplementary tables

**Supplementary Figure 1**

**
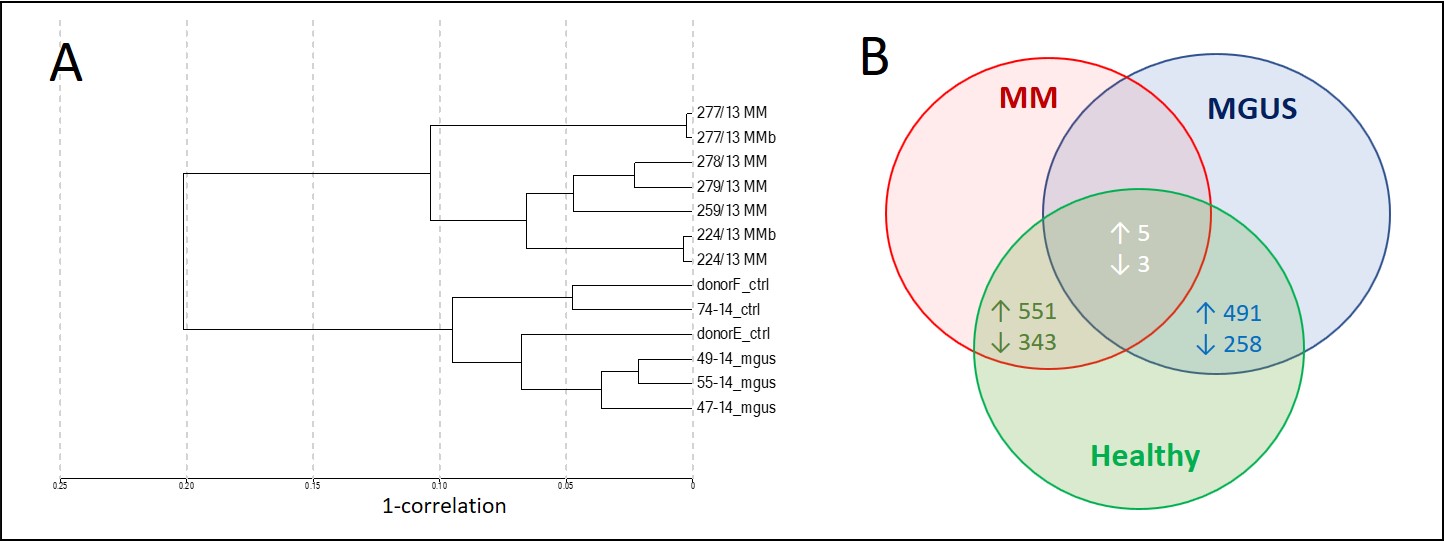
**Hierarchical clustering of gene expression for HDN obtained from MM, MGUS and healthy subjects (ctrl). MGUS- clusters separate from MM-HDN with a high degree of correlation to the MM-HDN, higher than ctrl.

**Supplementary Figure 2**

**Change in CD64 and CD16 expression on surface of normal HDN upon myeloma-related soluble factors**

**
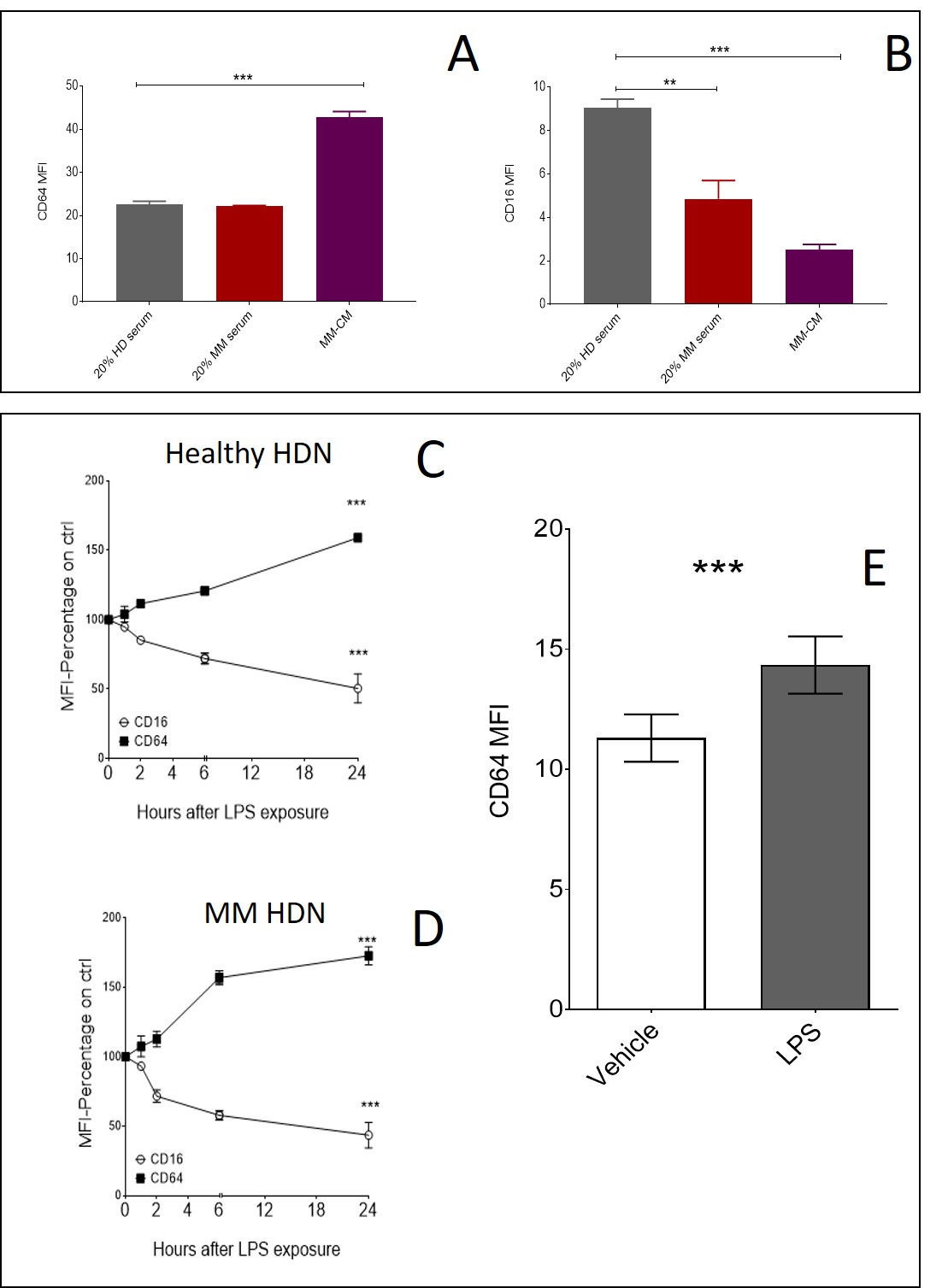
**

High-density neutrophils freshly isolated from healthy subjects were seeded in presence of 20%serum obtained from 8 MGUS and 8 MM patients or conditioned media of 2 human myeloma cell lines (MM1.s and U266) and mean of fluorescence intensity of CD64 **(A)** and CD16 **(B)** were assessed by flow cytometry 24 hours later, in three independent experiments. For more robust statistical evaluation, MFI values were converted to a resolution metric, such as the RD defined as (Median_treatment_-Median_control_)/(rSD_treatment_+rSD_control_) to further perform parametric tests to compare results of different experiments and runs. Each bar represents mean of three independent experiments. Data are evaluated using ANOVA test, significant p-value<0.005. In **(B)**, ANOVA test with post-hoc analysis was further used to compare MFI between two single groups.

Expression of CD64 **(C)** and CD16 **(D)** during LPS exposure in vitro overtime in HDN obtained from healthy, MGUS or MM donors is shown. Stars denote p-value (***, p<0.001, **, p<0.05) using two-way ANOVA test for repeated measures.

After 3 hours from LPS exposure, CD64 MFI in MM-HDN was significantly increased **(E)**, according to t-test**.**

**Supplementary Figure 3**

**HDN are immune-suppressive in MM and MGUS subjects**

**
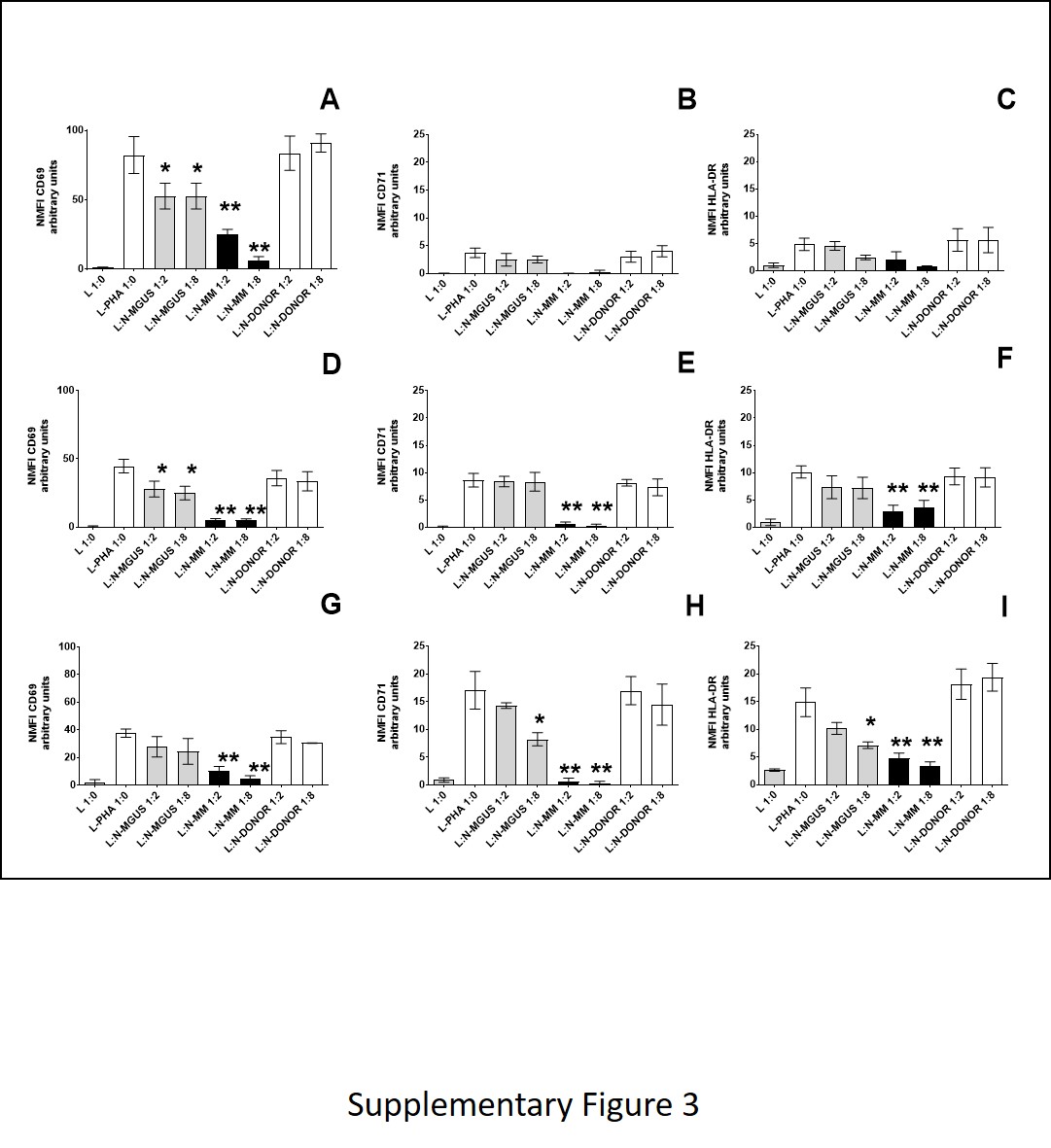
**

CD3^+^ T-cells from healthy donors were not activated or activated with PHA and co-cultured with purified neutrophils freshly isolated from MGUS/MM patients or healthy donors, matched for sex and age, at increasing concentration (L:N ratio 1:2, 1:8). After 24 (upper panel, A-B-C), 48 (middle panel, D-E-F) and 72 (lower panel, G-H-I) hours lymphocytes were examined for activation marker expression: CD69, CD71 and HLA-DR. Results are the MFI mean ± SD of duplicates from ten donors and ten patients, and are representative of eight independent experiments.

Stars denote p-value (***, p<0.001, *, p<0.05) using ANOVA-test and multiple comparisons.

Abbreviations: L: lymphocyte, N: neutrophils, PHA-P: phytohemagglutinin, SD: standard deviation.

**Supplementary Figure 4**

**Expression of MOSC1 in MGUS and MM HDN**

**
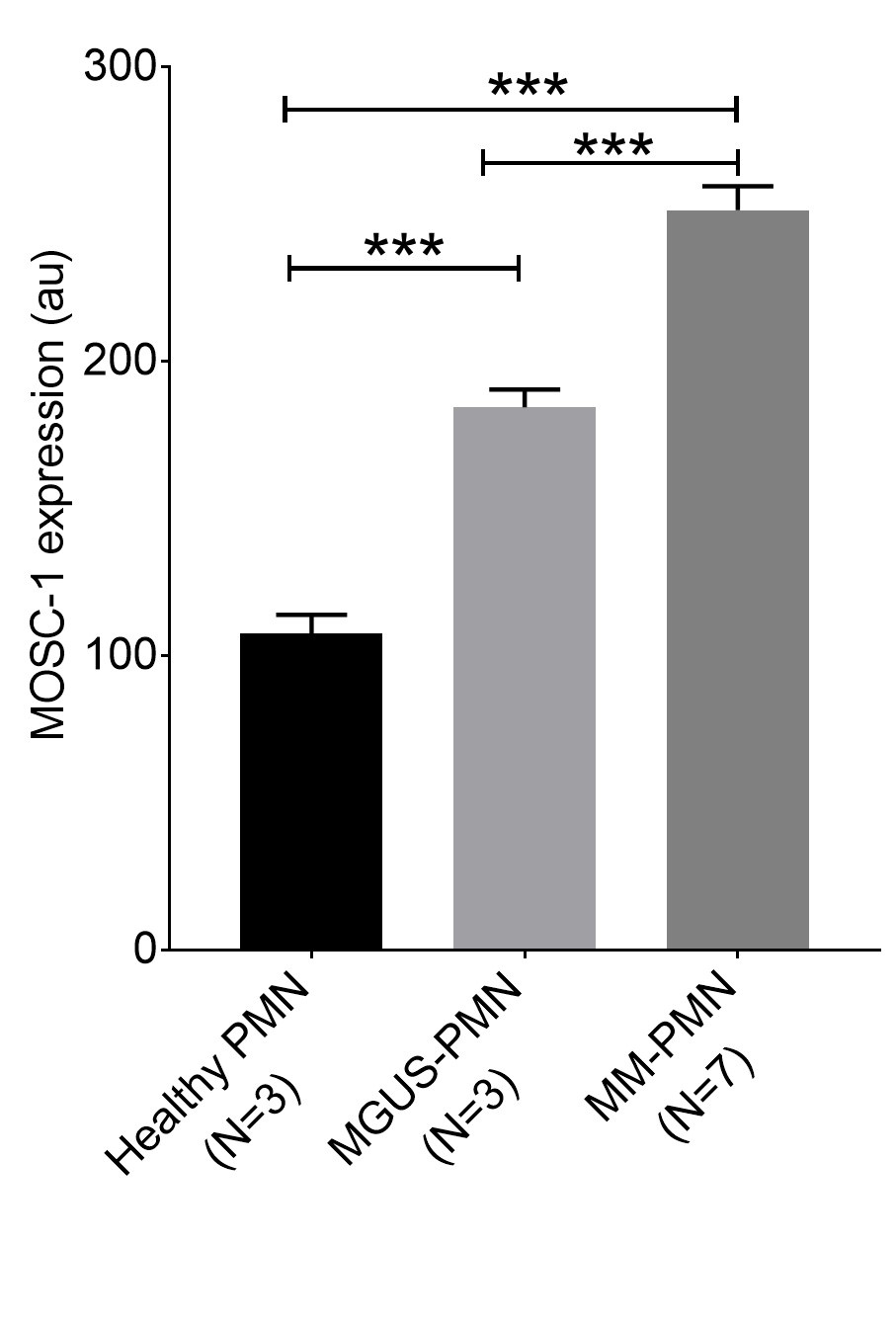
**

MOSC1, also known as MARC1 (mitochondrial amidoxime reducing component 1) is a component of an N-hydroxylated prodrug-converting complex, able to reduce Nω-hydroxy-L-arginine (NOHA) and Nω-hydroxy-Nδ-methyl-L-arginine (NHAM) in to L-arginine and Nδ-methyl-L-arginine, respectively. Its expression levels are increased in HDN obtained from MM and MGUS subjects, compared to healthy ones.

Stars denote p-value (***, p<0.001, *, p<0.05) using ANOVA-test and multiple comparisons.

**Supplementary Figure 5**

**Morphology of high-density neutrophils isolated from peripheral blood of multiple myeloma patients**

**
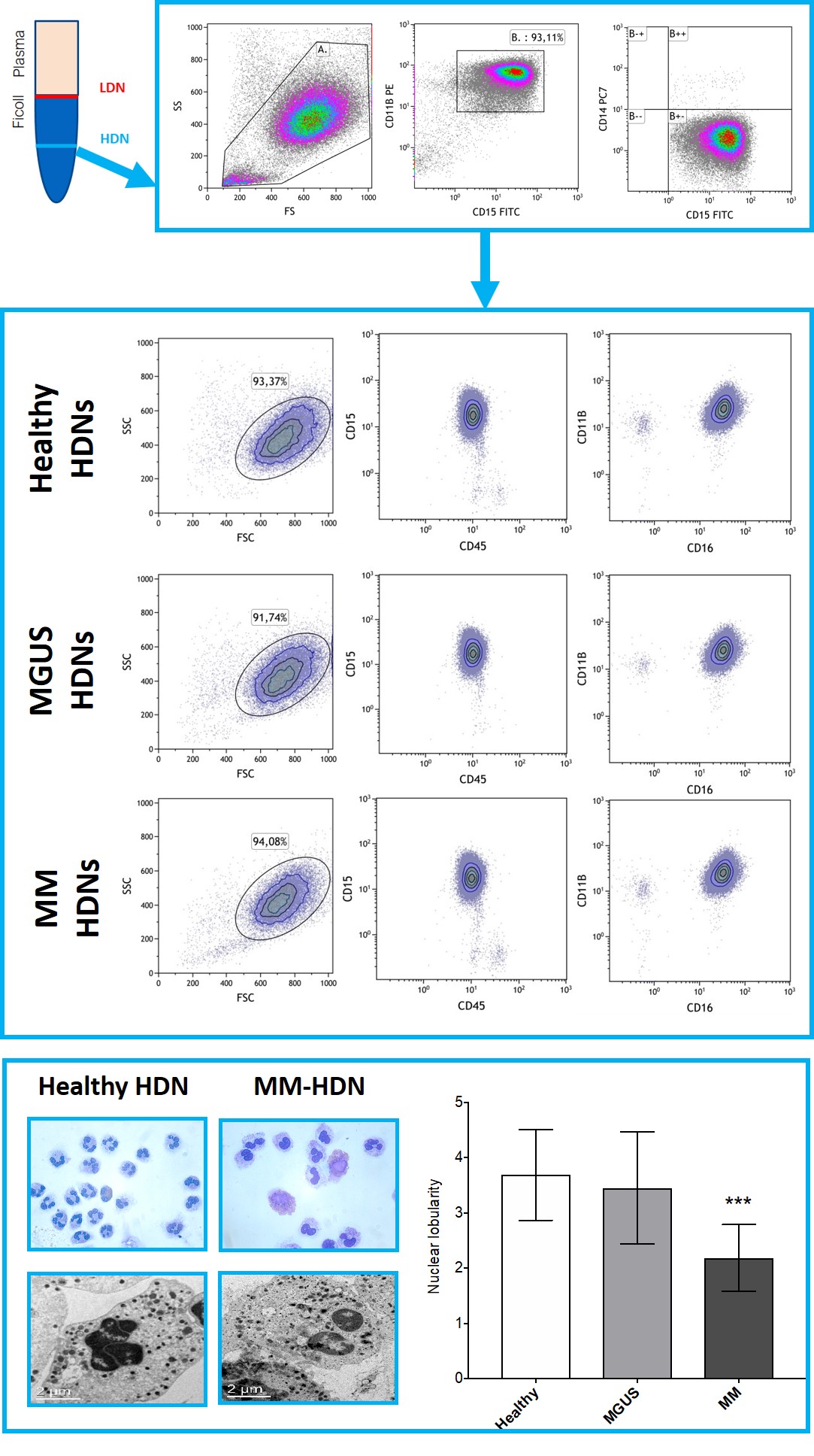
**

High density neutrophils (HDN) were isolated by immune-magnetic-based positive selection after density gradient sedimentation. HDNs were mature neutrophils identifiable as CD11b^+^CD16^+^ elements in the suspension. Morphology of HDN is shown for healthy and MM subjects as detected by optical (left panels and transmission electron microscopy. Nuclear lobularity, as detected by imaging analysis of 100 cells/sample, was progressively reduced in the progression from MGUS through MM showing a functional difference to depict with further studies.

To avoid a significant impact of contaminating cells on the results, the purity of HDN for functional assay (at least 95%) was assessed by flow cytometry to identify CD45^+^CD11b^+^CD15^+^CD14^-^ cells, as shown for a representative HDN sample.

Stars denote p-value (***, p<0.001, *, p<0.05) using ANOVA-test with post-hoc analysis.

**Supplementary Table 1**

**List of 551 up-regulated and 343 down-regulated genes in MM-HDN compared to healthy controls (p<0.0001)**

| **Up-regulated genes** | | | | | **Down-regulated genes** | | | | |
| --- | --- | --- | --- | --- | --- | --- | --- | --- | --- |
| **ENTREZ_GENE_ID** | **SYMBOL** | **FC** | **log**  **FC** | **p-value** | **ENTREZ_GENE_ID** | **SYMBOL** | **FC** | **log**  **FC** | **p-value** |
| 64407 | RGS18 | 6.86 | 2.78 | 0.03 | 100129267 | LOC100129267 | 0.79 | -0.34 | 0.02 |
| 60675 | PROK2 | 6.81 | 2.77 | 0.00 | 56985 | C17orf48 | 0.77 | -0.38 | 0.04 |
| 25801 | GCA | 6.62 | 2.73 | 0.00 | 341112 | LOC341112 | 0.76 | -0.39 | 0.04 |
| 23401 | FRAT2 | 6.57 | 2.71 | 0.00 | 84154 | RPF2 | 0.75 | -0.41 | 0.04 |
| 3310 | HSPA6 | 5.93 | 2.57 | 0.05 | 219927 | MRPL21 | 0.75 | -0.42 | 0.04 |
| 9123 | SLC16A3 | 5.44 | 2.44 | 0.04 | 160622 | GRASP | 0.74 | -0.43 | 0.02 |
| 3055 | HCK | 5.25 | 2.39 | 0.03 | 84868 | HAVCR2 | 0.73 | -0.45 | 0.01 |
| 26020 | LRP10 | 5.05 | 2.34 | 0.00 | 646574 | LOC646574 | 0.73 | -0.45 | 0.01 |
| 120425 | AMICA1 | 5.00 | 2.32 | 0.03 | 23119 | HIC2 | 0.73 | -0.46 | 0.04 |
| 8837 | CFLAR | 4.93 | 2.30 | 0.00 | 645737 | LOC645737 | 0.72 | -0.47 | 0.01 |
| 64844 | MARCH7 | 4.91 | 2.30 | 0.00 | 9855 | FARP2 | 0.72 | -0.47 | 0.04 |
| 116496 | FAM129A | 4.90 | 2.29 | 0.02 | 8754 | ADAM9 | 0.72 | -0.47 | 0.02 |
| 54518 | APBB1IP | 4.87 | 2.28 | 0.03 | 1514 | CTSL1 | 0.70 | -0.51 | 0.00 |
| 64333 | ARHGAP9 | 4.44 | 2.15 | 0.01 | 56271 | BEX4 | 0.70 | -0.51 | 0.04 |
| 5836 | PYGL | 4.21 | 2.08 | 0.04 | 64412 | GZF1 | 0.69 | -0.53 | 0.01 |
| 51734 | SEPX1 | 4.19 | 2.07 | 0.00 | 100129645 | LOC100129645 | 0.69 | -0.53 | 0.04 |
| 7791 | ZYX | 4.18 | 2.06 | 0.02 | 79159 | NOL12 | 0.69 | -0.53 | 0.03 |
| 2215 | FCGR3B | 4.14 | 2.05 | 0.02 | 51277 | DNAJC27 | 0.69 | -0.54 | 0.01 |
| 976 | CD97 | 4.11 | 2.04 | 0.00 | 84973 | SNHG7 | 0.69 | -0.54 | 0.03 |
| 1445 | CSK | 4.08 | 2.03 | 0.02 | 23753 | SDF2L1 | 0.69 | -0.54 | 0.04 |
| 55281 | TMEM140 | 3.93 | 1.98 | 0.02 | 122953 | JDP2 | 0.69 | -0.54 | 0.02 |
| 11237 | RNF24 | 3.85 | 1.95 | 0.00 | 92815 | HIST3H2A | 0.68 | -0.56 | 0.01 |
| 64386 | MMP25 | 3.83 | 1.94 | 0.02 | 8653 | DDX3Y | 0.68 | -0.56 | 0.00 |
| 83716 | CRISPLD2 | 3.75 | 1.91 | 0.00 | 6201 | RPS7 | 0.68 | -0.56 | 0.02 |
| 9750 | FAM65B | 3.73 | 1.90 | 0.02 | 5393 | EXOSC9 | 0.68 | -0.56 | 0.04 |
| 2319 | FLOT2 | 3.69 | 1.88 | 0.03 | 10362 | HMG20B | 0.67 | -0.57 | 0.05 |
| 64218 | SEMA4A | 3.68 | 1.88 | 0.04 | 1462 | VCAN | 0.67 | -0.57 | 0.01 |
| 2242 | FES | 3.67 | 1.87 | 0.03 | 100129566 | LOC100129566 | 0.67 | -0.57 | 0.01 |
| 29887 | SNX10 | 3.67 | 1.87 | 0.03 | 29105 | C16orf80 | 0.66 | -0.60 | 0.02 |
| 11314 | CD300A | 3.64 | 1.87 | 0.02 | 83939 | EIF2A | 0.66 | -0.60 | 0.01 |
| 5724 | PTAFR | 3.64 | 1.86 | 0.00 | 649839 | LOC649839 | 0.65 | -0.61 | 0.00 |
| 54502 | RBM47 | 3.63 | 1.86 | 0.03 | 973 | CD79A | 0.65 | -0.62 | 0.02 |
| 29982 | NRBF2 | 3.61 | 1.85 | 0.01 | 79706 | PRKRIP1 | 0.65 | -0.62 | 0.03 |
| 400818 | NBPF20 | 3.58 | 1.84 | 0.00 | 100134660 | LOC100134660 | 0.65 | -0.62 | 0.01 |
| 6809 | STX3 | 3.53 | 1.82 | 0.03 | 7798 | LUZP1 | 0.65 | -0.62 | 0.02 |
| 643313 | LOC643313 | 3.52 | 1.82 | 0.01 | 8106 | PABPN1 | 0.65 | -0.63 | 0.01 |
| 8650 | NUMB | 3.50 | 1.81 | 0.04 | 10236 | HNRPR | 0.64 | -0.64 | 0.04 |
| 79143 | MBOAT7 | 3.48 | 1.80 | 0.00 | 10482 | NXF1 | 0.64 | -0.64 | 0.04 |
| 10866 | HCP5 | 3.47 | 1.79 | 0.00 | 55330 | CNO | 0.64 | -0.64 | 0.01 |
| 121260 | SLC15A4 | 3.46 | 1.79 | 0.00 | 79078 | C1orf50 | 0.64 | -0.64 | 0.00 |
| 4688 | NCF2 | 3.45 | 1.79 | 0.02 | 652864 | LOC652864 | 0.64 | -0.65 | 0.00 |
| 9208 | LRRFIP1 | 3.45 | 1.79 | 0.00 | 56993 | TOMM22 | 0.64 | -0.65 | 0.00 |
| 9334 | B4GALT5 | 3.38 | 1.76 | 0.00 | 23180 | RFTN1 | 0.64 | -0.65 | 0.05 |
| 1318 | SLC31A2 | 3.38 | 1.76 | 0.04 | 64975 | MRPL41 | 0.63 | -0.66 | 0.04 |
| 55843 | ARHGAP15 | 3.37 | 1.75 | 0.04 | 653702 | LOC653702 | 0.63 | -0.67 | 0.01 |
| 201799 | TMEM154 | 3.37 | 1.75 | 0.04 | 653437 | AQP12B | 0.63 | -0.67 | 0.01 |
| 64859 | OBFC2A | 3.36 | 1.75 | 0.01 | 88745 | C6orf153 | 0.62 | -0.68 | 0.01 |
| 19 | ABCA1 | 3.31 | 1.72 | 0.04 | 646672 | LOC646672 | 0.62 | -0.68 | 0.02 |
| 253018 | HCG27 | 3.28 | 1.71 | 0.01 | 29919 | C18orf8 | 0.62 | -0.69 | 0.02 |
| 55793 | FAM63A | 3.28 | 1.71 | 0.00 | 387703 | LOC387703 | 0.62 | -0.69 | 0.00 |
| 8031 | NCOA4 | 3.25 | 1.70 | 0.03 | 6631 | SNRPC | 0.62 | -0.69 | 0.00 |
| 338442 | GPR109A | 3.23 | 1.69 | 0.00 | 55159 | RFWD3 | 0.62 | -0.70 | 0.01 |
| 55577 | NAGK | 3.22 | 1.69 | 0.02 | 441763 | LOC441763 | 0.61 | -0.71 | 0.03 |
| 2313 | FLI1 | 3.22 | 1.69 | 0.05 | 64061 | TSPYL2 | 0.61 | -0.71 | 0.05 |
| 1052 | CEBPD | 3.19 | 1.68 | 0.00 | 2791 | GNG11 | 0.61 | -0.72 | 0.00 |
| 3759 | KCNJ2 | 3.18 | 1.67 | 0.03 | 56929 | FEM1C | 0.60 | -0.73 | 0.02 |
| 728841 | NBPF8 | 3.16 | 1.66 | 0.01 | 7416 | VDAC1 | 0.60 | -0.74 | 0.00 |
| 114785 | MBD6 | 3.16 | 1.66 | 0.05 | 6147 | RPL23A | 0.60 | -0.74 | 0.03 |
| 58526 | MID1IP1 | 3.12 | 1.64 | 0.03 | 51335 | NGRN | 0.60 | -0.74 | 0.01 |
| 23558 | WBP2 | 3.11 | 1.64 | 0.00 | 390735 | LOC390735 | 0.60 | -0.75 | 0.04 |
| 56882 | CDC42SE1 | 3.09 | 1.63 | 0.00 | 5702 | PSMC3 | 0.59 | -0.75 | 0.05 |
| 202309 | GAPT | 3.08 | 1.62 | 0.00 | 442270 | LOC442270 | 0.59 | -0.76 | 0.02 |
| 7130 | TNFAIP6 | 3.06 | 1.61 | 0.05 | 646909 | LOC646909 | 0.59 | -0.76 | 0.01 |
| 730820 | LOC730820 | 3.03 | 1.60 | 0.04 | 10020 | GNE | 0.59 | -0.76 | 0.02 |
| 6992 | PPP1R11 | 3.01 | 1.59 | 0.00 | 164 | AP1G1 | 0.59 | -0.76 | 0.04 |
| 7409 | VAV1 | 3.00 | 1.58 | 0.00 | 100130715 | LOC100130715 | 0.59 | -0.77 | 0.01 |
| 256586 | LYSMD2 | 2.99 | 1.58 | 0.05 | 6675 | UAP1 | 0.58 | -0.77 | 0.05 |
| 140885 | SIRPA | 2.99 | 1.58 | 0.01 | 90809 | TMEM55B | 0.58 | -0.78 | 0.00 |
| 6404 | SELPLG | 2.98 | 1.57 | 0.00 | 644423 | LOC644423 | 0.58 | -0.78 | 0.03 |
| 153222 | C5orf41 | 2.98 | 1.57 | 0.01 | 100151683 | RNU4ATAC | 0.58 | -0.79 | 0.00 |
| 523 | ATP6V1A | 2.97 | 1.57 | 0.00 | 51188 | SS18L2 | 0.58 | -0.79 | 0.05 |
| 9170 | EDG4 | 2.97 | 1.57 | 0.00 | 4809 | NHP2L1 | 0.58 | -0.79 | 0.03 |
| 5899 | RALB | 2.97 | 1.57 | 0.00 | 100130561 | LOC100130561 | 0.58 | -0.80 | 0.02 |
| 9061 | PAPSS1 | 2.96 | 1.57 | 0.00 | 100132715 | LOC100132715 | 0.57 | -0.80 | 0.04 |
| 6688 | SPI1 | 2.96 | 1.57 | 0.00 | 100131526 | LOC100131526 | 0.57 | -0.81 | 0.04 |
| 10287 | RGS19 | 2.96 | 1.56 | 0.00 | 550643 | LOC550643 | 0.57 | -0.81 | 0.03 |
| 6778 | STAT6 | 2.95 | 1.56 | 0.02 | 2815 | GP9 | 0.56 | -0.82 | 0.01 |
| 26578 | OSTF1 | 2.95 | 1.56 | 0.05 | 2107 | ETF1 | 0.56 | -0.84 | 0.03 |
| 89849 | ATG16L2 | 2.94 | 1.56 | 0.04 | 148479 | PHF13 | 0.55 | -0.85 | 0.01 |
| 1955 | MEGF9 | 2.94 | 1.56 | 0.03 | 64145 | ZFYVE20 | 0.55 | -0.86 | 0.00 |
| 2867 | FFAR2 | 2.93 | 1.55 | 0.00 | 51647 | FAM96B | 0.55 | -0.87 | 0.00 |
| 79650 | C16orf57 | 2.92 | 1.55 | 0.04 | 55839 | CENPN | 0.55 | -0.87 | 0.00 |
| 11031 | RAB31 | 2.91 | 1.54 | 0.03 | 6612 | SUMO3 | 0.55 | -0.87 | 0.02 |
| 88523 | LOC88523 | 2.91 | 1.54 | 0.05 | 2950 | GSTP1 | 0.54 | -0.88 | 0.01 |
| 6890 | TAP1 | 2.90 | 1.54 | 0.01 | 79759 | ZNF668 | 0.54 | -0.88 | 0.00 |
| 7132 | TNFRSF1A | 2.88 | 1.52 | 0.03 | 8668 | EIF3I | 0.54 | -0.88 | 0.03 |
| 929 | CD14 | 2.88 | 1.52 | 0.00 | 374291 | NDUFS7 | 0.54 | -0.88 | 0.00 |
| 29065 | ASAP1IT1 | 2.87 | 1.52 | 0.00 | 389286 | LOC389286 | 0.54 | -0.89 | 0.00 |
| 8698 | S1PR4 | 2.86 | 1.52 | 0.01 | 52 | ACP1 | 0.54 | -0.89 | 0.01 |
| 399748 | LOC399748 | 2.85 | 1.51 | 0.03 | 1973 | EIF4A1 | 0.54 | -0.89 | 0.01 |
| 158747 | MOSPD2 | 2.84 | 1.50 | 0.02 | 136143 | LOC136143 | 0.54 | -0.90 | 0.01 |
| 404636 | FAM45A | 2.80 | 1.48 | 0.01 | 155370 | SBDSP | 0.54 | -0.90 | 0.00 |
| 11027 | LILRA2 | 2.78 | 1.48 | 0.00 | 391019 | LOC391019 | 0.53 | -0.91 | 0.03 |
| 57515 | SERINC1 | 2.77 | 1.47 | 0.04 | 51082 | POLR1D | 0.53 | -0.91 | 0.04 |
| 604 | BCL6 | 2.74 | 1.46 | 0.00 | 646849 | LOC646849 | 0.53 | -0.91 | 0.01 |
| 9368 | SLC9A3R1 | 2.74 | 1.45 | 0.01 | 5714 | PSMD8 | 0.53 | -0.92 | 0.00 |
| 29978 | UBQLN2 | 2.74 | 1.45 | 0.02 | 51061 | TXNDC11 | 0.53 | -0.92 | 0.02 |
| 727908 | LOC727908 | 2.73 | 1.45 | 0.02 | 84844 | PHF5A | 0.53 | -0.92 | 0.01 |
| 80223 | RAB11FIP1 | 2.71 | 1.44 | 0.01 | 255783 | LOC255783 | 0.53 | -0.93 | 0.03 |
| 4542 | MYO1F | 2.69 | 1.43 | 0.02 | 387791 | LOC387791 | 0.53 | -0.93 | 0.00 |
| 10097 | ACTR2 | 2.68 | 1.42 | 0.00 | 645693 | LOC645693 | 0.52 | -0.94 | 0.00 |
| 23569 | PADI4 | 2.64 | 1.40 | 0.03 | 4715 | NDUFB9 | 0.52 | -0.94 | 0.01 |
| 5110 | PCMT1 | 2.64 | 1.40 | 0.02 | 60314 | C12orf10 | 0.52 | -0.95 | 0.00 |
| 7431 | VIM | 2.64 | 1.40 | 0.00 | 29889 | GNL2 | 0.52 | -0.95 | 0.00 |
| 143 | PARP4 | 2.62 | 1.39 | 0.01 | 6628 | SNRPB | 0.52 | -0.96 | 0.01 |
| 392 | ARHGAP1 | 2.60 | 1.38 | 0.03 | 116541 | MRPL54 | 0.51 | -0.96 | 0.03 |
| 84803 | AGPAT9 | 2.60 | 1.38 | 0.02 | 6427 | SFRS2 | 0.51 | -0.97 | 0.00 |
| 6813 | STXBP2 | 2.59 | 1.37 | 0.00 | 26834 | RNU4-2 | 0.51 | -0.97 | 0.03 |
| 23506 | KIAA0240 | 2.59 | 1.37 | 0.04 | 51503 | CWC15 | 0.51 | -0.98 | 0.02 |
| 81857 | MED25 | 2.58 | 1.37 | 0.02 | 51398 | C19orf56 | 0.51 | -0.98 | 0.01 |
| 9170 | LPAR2 | 2.57 | 1.36 | 0.02 | 83460 | TMEM93 | 0.50 | -0.99 | 0.02 |
| 1138 | CHRNA5 | 2.57 | 1.36 | 0.00 | 389672 | LOC389672 | 0.50 | -1.00 | 0.01 |
| 4947 | OAZ2 | 2.55 | 1.35 | 0.00 | 100128731 | LOC100128731 | 0.50 | -1.00 | 0.01 |
| 100133516 | LOC100133516 | 2.53 | 1.34 | 0.00 | 727826 | LOC727826 | 0.49 | -1.02 | 0.04 |
| 730278 | LOC730278 | 2.53 | 1.34 | 0.03 | 378805 | FLJ43663 | 0.49 | -1.02 | 0.00 |
| 222194 | RSBN1L | 2.52 | 1.33 | 0.04 | 200185 | KRTCAP2 | 0.49 | -1.02 | 0.00 |
| 2124 | EVI2B | 2.52 | 1.33 | 0.02 | 10572 | SIVA | 0.49 | -1.03 | 0.01 |
| 1831 | TSC22D3 | 2.52 | 1.33 | 0.00 | 387066 | C6orf160 | 0.49 | -1.03 | 0.00 |
| 23550 | PSD4 | 2.50 | 1.32 | 0.01 | 10357 | HMGB1L1 | 0.49 | -1.04 | 0.00 |
| 22878 | KIAA1012 | 2.50 | 1.32 | 0.02 | 5204 | PFDN5 | 0.49 | -1.04 | 0.01 |
| 23304 | UBR2 | 2.49 | 1.32 | 0.03 | 6170 | RPL39 | 0.49 | -1.04 | 0.03 |
| 121274 | ZNF641 | 2.49 | 1.31 | 0.00 | 27335 | EIF3K | 0.49 | -1.04 | 0.02 |
| 23013 | SPEN | 2.48 | 1.31 | 0.00 | 641768 | LOC641768 | 0.49 | -1.04 | 0.05 |
| 5728 | PTEN | 2.47 | 1.31 | 0.04 | 729646 | LOC729646 | 0.48 | -1.05 | 0.00 |
| 5236 | PGM1 | 2.46 | 1.30 | 0.00 | 539 | ATP5O | 0.48 | -1.05 | 0.02 |
| 84109 | QRFPR | 2.45 | 1.30 | 0.00 | 729500 | LOC729500 | 0.48 | -1.06 | 0.00 |
| 201176 | ARHGAP27 | 2.45 | 1.29 | 0.04 | 1915 | EEF1A1 | 0.48 | -1.06 | 0.01 |
| 8291 | DYSF | 2.44 | 1.29 | 0.04 | 26823 | RNU12 | 0.47 | -1.08 | 0.00 |
| 7454 | WAS | 2.43 | 1.28 | 0.00 | 730235 | LOC730235 | 0.47 | -1.08 | 0.02 |
| 23523 | CABIN1 | 2.43 | 1.28 | 0.02 | 647673 | LOC647673 | 0.47 | -1.08 | 0.00 |
| 26030 | PLEKHG3 | 2.43 | 1.28 | 0.02 | 3608 | ILF2 | 0.47 | -1.09 | 0.00 |
| 54918 | CMTM6 | 2.43 | 1.28 | 0.03 | 728791 | LOC728791 | 0.47 | -1.09 | 0.00 |
| 7620 | ZNF69 | 2.41 | 1.27 | 0.00 | 81576 | CCDC130 | 0.47 | -1.09 | 0.00 |
| 54820 | NDE1 | 2.41 | 1.27 | 0.00 | 387753 | LOC387753 | 0.47 | -1.10 | 0.01 |
| 3920 | LAMP2 | 2.41 | 1.27 | 0.05 | 100131866 | LOC100131866 | 0.47 | -1.10 | 0.01 |
| 54583 | EGLN1 | 2.39 | 1.26 | 0.02 | 730288 | LOC730288 | 0.46 | -1.11 | 0.04 |
| 53917 | RAB24 | 2.39 | 1.26 | 0.04 | 642817 | LOC642817 | 0.46 | -1.11 | 0.02 |
| 57498 | KIDINS220 | 2.39 | 1.26 | 0.03 | 285958 | C7orf40 | 0.46 | -1.13 | 0.00 |
| 653361 | NCF1 | 2.37 | 1.24 | 0.02 | 7277 | TUBA4A | 0.46 | -1.13 | 0.01 |
| 301 | ANXA1 | 2.37 | 1.24 | 0.00 | 10078 | TSSC4 | 0.45 | -1.15 | 0.00 |
| 2268 | FGR | 2.36 | 1.24 | 0.05 | 440176 | RPL12P6 | 0.45 | -1.15 | 0.00 |
| 8930 | MBD4 | 2.36 | 1.24 | 0.00 | 27125 | AFF4 | 0.45 | -1.15 | 0.01 |
| 3101 | HK3 | 2.35 | 1.23 | 0.00 | 51119 | SBDS | 0.45 | -1.16 | 0.00 |
| 728519 | LOC728519 | 2.34 | 1.23 | 0.04 | 100131787 | LOC100131787 | 0.44 | -1.18 | 0.04 |
| 55233 | MOBKL1B | 2.34 | 1.23 | 0.00 | 341315 | LOC341315 | 0.44 | -1.18 | 0.03 |
| 54502 | FLJ20273 | 2.34 | 1.22 | 0.02 | 26831 | RNU5A | 0.44 | -1.19 | 0.00 |
| 26118 | WSB1 | 2.34 | 1.22 | 0.00 | 2483 | FRG1 | 0.44 | -1.19 | 0.00 |
| 23214 | XPO6 | 2.33 | 1.22 | 0.02 | 2197 | FAU | 0.44 | -1.20 | 0.02 |
| 23765 | IL17RA | 2.33 | 1.22 | 0.00 | 10208 | USPL1 | 0.43 | -1.21 | 0.01 |
| 170954 | KIAA1949 | 2.33 | 1.22 | 0.03 | 388720 | LOC388720 | 0.43 | -1.21 | 0.00 |
| 5777 | PTPN6 | 2.33 | 1.22 | 0.02 | 100130553 | LOC100130553 | 0.43 | -1.23 | 0.01 |
| 7516 | XRCC2 | 2.31 | 1.21 | 0.01 | 5473 | PPBP | 0.42 | -1.23 | 0.00 |
| 23211 | ZC3H4 | 2.31 | 1.21 | 0.03 | 440733 | LOC440733 | 0.42 | -1.25 | 0.04 |
| 55147 | RBM23 | 2.30 | 1.20 | 0.00 | 689 | BTF3 | 0.42 | -1.25 | 0.04 |
| 160728 | SLC5A8 | 2.30 | 1.20 | 0.00 | 100008588 | LOC100008588 | 0.42 | -1.25 | 0.01 |
| 11120 | BTN2A1 | 2.28 | 1.19 | 0.01 | 819 | CAMLG | 0.42 | -1.26 | 0.00 |
| 58190 | CTDSP1 | 2.27 | 1.18 | 0.01 | 51759 | C9orf78 | 0.42 | -1.26 | 0.00 |
| 4214 | MAP3K1 | 2.27 | 1.18 | 0.00 | 140032 | RPS4Y2 | 0.42 | -1.27 | 0.00 |
| 653157 | LOC653157 | 2.27 | 1.18 | 0.00 | 442454 | LOC442454 | 0.41 | -1.27 | 0.00 |
| 3192 | HNRNPU | 2.26 | 1.18 | 0.03 | 6232 | RPS27 | 0.41 | -1.29 | 0.03 |
| 100130886 | LOC100130886 | 2.26 | 1.18 | 0.05 | 293 | SLC25A6 | 0.41 | -1.30 | 0.01 |
| 25764 | C15orf63 | 2.26 | 1.17 | 0.04 | 8667 | EIF3H | 0.41 | -1.30 | 0.00 |
| 1871 | E2F3 | 2.25 | 1.17 | 0.00 | 6139 | RPL17 | 0.41 | -1.30 | 0.00 |
| 100128460 | LOC100128460 | 2.24 | 1.17 | 0.00 | 51187 | RSL24D1 | 0.41 | -1.30 | 0.00 |
| 4055 | LTBR | 2.24 | 1.16 | 0.04 | 6160 | RPL31 | 0.41 | -1.30 | 0.01 |
| 57477 | SHROOM4 | 2.23 | 1.16 | 0.00 | 514 | ATP5E | 0.41 | -1.30 | 0.01 |
| 9181 | ARHGEF2 | 2.23 | 1.16 | 0.00 | 644937 | LOC644937 | 0.40 | -1.31 | 0.00 |
| 830 | CAPZA2 | 2.23 | 1.16 | 0.02 | 54107 | POLE3 | 0.40 | -1.31 | 0.01 |
| 10096 | ACTR3 | 2.23 | 1.16 | 0.03 | 100133931 | LOC100133931 | 0.40 | -1.31 | 0.01 |
| 2782 | GNB1 | 2.22 | 1.15 | 0.00 | 10519 | CIB1 | 0.40 | -1.31 | 0.00 |
| 23031 | MAST3 | 2.21 | 1.15 | 0.03 | 391777 | LOC391777 | 0.40 | -1.33 | 0.02 |
| 8993 | PGLYRP1 | 2.21 | 1.15 | 0.03 | 7388 | UQCRH | 0.40 | -1.34 | 0.01 |
| 84278 | HIATL2 | 2.20 | 1.14 | 0.00 | 401206 | LOC401206 | 0.40 | -1.34 | 0.00 |
| 6752 | SSTR2 | 2.18 | 1.13 | 0.00 | 649150 | LOC649150 | 0.40 | -1.34 | 0.01 |
| 154791 | HSPC268 | 2.18 | 1.13 | 0.00 | 389223 | LOC389223 | 0.39 | -1.34 | 0.00 |
| 4780 | NFE2L2 | 2.18 | 1.12 | 0.00 | 6229 | RPS24 | 0.39 | -1.35 | 0.00 |
| 64757 | MOSC1 | 2.18 | 1.12 | 0.00 | 6135 | RPL11 | 0.39 | -1.35 | 0.01 |
| 55033 | FKBP14 | 2.17 | 1.12 | 0.02 | 729279 | LOC729279 | 0.39 | -1.35 | 0.04 |
| 25797 | QPCT | 2.17 | 1.12 | 0.05 | 6205 | RPS11 | 0.39 | -1.36 | 0.03 |
| 64397 | ZFP106 | 2.16 | 1.11 | 0.00 | 6233 | RPS27A | 0.38 | -1.38 | 0.00 |
| 56203 | LMOD3 | 2.16 | 1.11 | 0.00 | 100129650 | LOC100129650 | 0.38 | -1.38 | 0.02 |
| 79134 | TMEM185B | 2.16 | 1.11 | 0.04 | 6156 | RPL30 | 0.38 | -1.39 | 0.00 |
| 226 | ALDOA | 2.16 | 1.11 | 0.01 | 728484 | LOC728484 | 0.38 | -1.39 | 0.03 |
| 9445 | ITM2B | 2.16 | 1.11 | 0.01 | 440991 | LOC440991 | 0.38 | -1.39 | 0.04 |
| 10627 | MYL12A | 2.15 | 1.11 | 0.02 | 1327 | COX4I1 | 0.38 | -1.39 | 0.00 |
| 257106 | ARHGAP30 | 2.15 | 1.10 | 0.03 | 3313 | HSPA9 | 0.38 | -1.40 | 0.00 |
| 10550 | ARL6IP5 | 2.15 | 1.10 | 0.00 | 648210 | LOC648210 | 0.38 | -1.40 | 0.02 |
| 80867 | HCG2P7 | 2.15 | 1.10 | 0.00 | 6181 | RPLP2 | 0.38 | -1.41 | 0.00 |
| 4856 | NOV | 2.14 | 1.10 | 0.01 | 338758 | LOC338758 | 0.38 | -1.41 | 0.00 |
| 391 | RHOG | 2.14 | 1.10 | 0.01 | 642210 | LOC642210 | 0.38 | -1.41 | 0.00 |
| 8439 | NSMAF | 2.13 | 1.09 | 0.03 | 648771 | LOC648771 | 0.38 | -1.41 | 0.00 |
| 25844 | YIPF3 | 2.13 | 1.09 | 0.00 | 728576 | LOC728576 | 0.37 | -1.42 | 0.00 |
| 55529 | TMEM55A | 2.12 | 1.09 | 0.01 | 441032 | EEF1AL7 | 0.37 | -1.42 | 0.00 |
| 51100 | SH3GLB1 | 2.12 | 1.08 | 0.00 | 100133812 | LOC100133812 | 0.37 | -1.42 | 0.01 |
| 734 | OSGIN2 | 2.12 | 1.08 | 0.01 | 402251 | LOC402251 | 0.37 | -1.43 | 0.00 |
| 79693 | YRDC | 2.12 | 1.08 | 0.00 | 645688 | LOC645688 | 0.37 | -1.43 | 0.00 |
| 7508 | XPC | 2.12 | 1.08 | 0.03 | 100130446 | LOC100130446 | 0.37 | -1.43 | 0.00 |
| 374403 | TBC1D10C | 2.12 | 1.08 | 0.01 | 440567 | UQCRHL | 0.37 | -1.44 | 0.00 |
| 100128274 | LOC100128274 | 2.11 | 1.08 | 0.01 | 6230 | RPS25 | 0.37 | -1.45 | 0.00 |
| 4689 | NCF4 | 2.11 | 1.08 | 0.03 | 387930 | LOC387930 | 0.36 | -1.46 | 0.02 |
| 23433 | RHOQ | 2.10 | 1.07 | 0.03 | 441034 | LOC441034 | 0.36 | -1.47 | 0.00 |
| 29924 | EPN1 | 2.09 | 1.07 | 0.02 | 6189 | RPS3A | 0.36 | -1.48 | 0.04 |
| 51478 | HSD17B7 | 2.09 | 1.06 | 0.00 | 649821 | LOC649821 | 0.36 | -1.48 | 0.02 |
| 355 | FAS | 2.08 | 1.06 | 0.02 | 284393 | LOC284393 | 0.36 | -1.49 | 0.02 |
| 9070 | ASH2L | 2.08 | 1.05 | 0.04 | 56942 | C16orf61 | 0.35 | -1.49 | 0.00 |
| 23505 | TMEM131 | 2.07 | 1.05 | 0.02 | 100133372 | LOC100133372 | 0.35 | -1.50 | 0.00 |
| 4051 | CYP4F3 | 2.07 | 1.05 | 0.04 | 643863 | LOC643863 | 0.35 | -1.50 | 0.04 |
| 2180 | ACSL1 | 2.06 | 1.04 | 0.01 | 389435 | LOC389435 | 0.35 | -1.51 | 0.00 |
| 80325 | ABTB1 | 2.06 | 1.04 | 0.02 | 391126 | LOC391126 | 0.35 | -1.51 | 0.03 |
| 51291 | GMIP | 2.05 | 1.03 | 0.00 | 9045 | RPL14 | 0.35 | -1.51 | 0.04 |
| 83719 | YPEL3 | 2.04 | 1.03 | 0.00 | 6210 | RPS15A | 0.35 | -1.52 | 0.00 |
| 643882 | LOC643882 | 2.04 | 1.03 | 0.01 | 729340 | LOC729340 | 0.35 | -1.52 | 0.03 |
| 84334 | C14orf153 | 2.04 | 1.03 | 0.02 | 650276 | LOC650276 | 0.35 | -1.52 | 0.04 |
| 2153 | F5 | 2.04 | 1.03 | 0.04 | 644068 | MGC87895 | 0.35 | -1.52 | 0.01 |
| 11275 | KLHL2 | 2.03 | 1.02 | 0.04 | 6208 | RPS14 | 0.35 | -1.53 | 0.00 |
| 401098 | LOC401098 | 2.03 | 1.02 | 0.00 | 100130980 | LOC100130980 | 0.34 | -1.57 | 0.00 |
| 136319 | MTPN | 2.03 | 1.02 | 0.04 | 6224 | RPS20 | 0.34 | -1.57 | 0.00 |
| 651309 | LOC651309 | 2.03 | 1.02 | 0.03 | 728820 | LOC728820 | 0.34 | -1.58 | 0.02 |
| 440704 | LOC440704 | 2.03 | 1.02 | 0.00 | 100129141 | LOC100129141 | 0.34 | -1.58 | 0.01 |
| 88455 | ANKRD13A | 2.03 | 1.02 | 0.02 | 643949 | LOC643949 | 0.33 | -1.58 | 0.00 |
| 100132585 | LOC100132585 | 2.03 | 1.02 | 0.00 | 643358 | LOC643358 | 0.33 | -1.58 | 0.02 |
| 867 | CBL | 2.02 | 1.02 | 0.02 | 648000 | LOC648000 | 0.33 | -1.58 | 0.04 |
| 404093 | CUEDC1 | 2.02 | 1.01 | 0.04 | 10399 | GNB2L1 | 0.33 | -1.59 | 0.00 |
| 5586 | PKN2 | 2.02 | 1.01 | 0.04 | 6218 | RPS17 | 0.33 | -1.59 | 0.01 |
| 126014 | OSCAR | 2.02 | 1.01 | 0.00 | 653232 | LOC653232 | 0.33 | -1.59 | 0.03 |
| 56255 | TMX4 | 2.01 | 1.01 | 0.01 | 400963 | LOC400963 | 0.33 | -1.60 | 0.03 |
| 57136 | C20orf3 | 2.01 | 1.01 | 0.00 | 645138 | LOC645138 | 0.33 | -1.60 | 0.00 |
| 81622 | UNC93B1 | 2.01 | 1.01 | 0.01 | 9929 | JOSD1 | 0.33 | -1.60 | 0.00 |
| 9051 | PSTPIP1 | 2.00 | 1.00 | 0.00 | 6157 | RPL27A | 0.33 | -1.61 | 0.00 |
| 90231 | KIAA2013 | 2.00 | 1.00 | 0.00 | 91561 | LOC91561 | 0.33 | -1.61 | 0.01 |
| 100129034 | LOC100129034 | 2.00 | 1.00 | 0.02 | 729402 | LOC729402 | 0.33 | -1.61 | 0.01 |
| 170370 | C10orf73 | 1.99 | 0.99 | 0.00 | 6207 | RPS13 | 0.33 | -1.62 | 0.01 |
| 7317 | UBA1 | 1.99 | 0.99 | 0.02 | 6204 | RPS10 | 0.33 | -1.62 | 0.00 |
| 9111 | NMI | 1.99 | 0.99 | 0.00 | 100129158 | LOC100129158 | 0.33 | -1.62 | 0.03 |
| 5880 | RAC2 | 1.99 | 0.99 | 0.02 | 6136 | RPL12 | 0.32 | -1.64 | 0.00 |
| 10025 | MED16 | 1.98 | 0.99 | 0.00 | 6217 | RPS16 | 0.32 | -1.64 | 0.00 |
| 126364 | LRRC25 | 1.98 | 0.98 | 0.02 | 730004 | LOC730004 | 0.32 | -1.64 | 0.04 |
| 8843 | GPR109B | 1.98 | 0.98 | 0.05 | 388474 | LOC388474 | 0.32 | -1.64 | 0.00 |
| 5894 | RAF1 | 1.98 | 0.98 | 0.03 | 100132795 | LOC100132795 | 0.32 | -1.65 | 0.00 |
| 51449 | PCYOX1 | 1.97 | 0.98 | 0.04 | 644039 | LOC644039 | 0.32 | -1.65 | 0.00 |
| 55924 | C1orf183 | 1.97 | 0.98 | 0.01 | 10153 | CEBPZ | 0.32 | -1.66 | 0.00 |
| 200942 | KLHDC8B | 1.97 | 0.98 | 0.02 | 729208 | LOC729208 | 0.32 | -1.66 | 0.00 |
| 7706 | TRIM25 | 1.97 | 0.98 | 0.03 | 6165 | RPL35A | 0.32 | -1.67 | 0.00 |
| 6573 | SLC19A1 | 1.97 | 0.98 | 0.02 | 648622 | LOC648622 | 0.31 | -1.67 | 0.01 |
| 142679 | DUSP19 | 1.97 | 0.97 | 0.00 | 6155 | RPL27 | 0.31 | -1.68 | 0.00 |
| 366 | AQP9 | 1.96 | 0.97 | 0.02 | 645683 | LOC645683 | 0.31 | -1.69 | 0.01 |
| 55 | ACPP | 1.96 | 0.97 | 0.01 | 6176 | RPLP1 | 0.31 | -1.69 | 0.01 |
| 345757 | FAM174A | 1.96 | 0.97 | 0.00 | 728658 | LOC728658 | 0.31 | -1.69 | 0.00 |
| 84888 | SPPL2A | 1.96 | 0.97 | 0.01 | 441876 | LOC441876 | 0.31 | -1.70 | 0.02 |
| 10487 | CAP1 | 1.95 | 0.97 | 0.02 | 220433 | LOC220433 | 0.31 | -1.70 | 0.00 |
| 53346 | TM6SF1 | 1.95 | 0.96 | 0.03 | 100131196 | LOC100131196 | 0.31 | -1.71 | 0.00 |
| 57161 | PELI2 | 1.95 | 0.96 | 0.03 | 730187 | LOC730187 | 0.30 | -1.72 | 0.01 |
| 84984 | C3orf34 | 1.94 | 0.96 | 0.00 | 646195 | LOC646195 | 0.30 | -1.75 | 0.02 |
| 100129211 | LOC100129211 | 1.94 | 0.96 | 0.02 | 645387 | LOC645387 | 0.30 | -1.75 | 0.01 |
| 3840 | KPNA4 | 1.93 | 0.95 | 0.03 | 100127993 | LOC100127993 | 0.30 | -1.76 | 0.01 |
| 6737 | TRIM21 | 1.93 | 0.95 | 0.04 | 343184 | LOC343184 | 0.29 | -1.77 | 0.01 |
| 64789 | DEM1 | 1.93 | 0.95 | 0.03 | 729742 | LOC729742 | 0.29 | -1.77 | 0.02 |
| 57132 | CHMP1B | 1.93 | 0.95 | 0.00 | 652071 | LOC652071 | 0.29 | -1.77 | 0.02 |
| 9748 | SLK | 1.93 | 0.95 | 0.01 | 645899 | LOC645899 | 0.29 | -1.78 | 0.04 |
| 818 | CAMK2G | 1.93 | 0.95 | 0.01 | 643433 | LOC643433 | 0.29 | -1.78 | 0.02 |
| 4067 | LYN | 1.92 | 0.94 | 0.04 | 440927 | LOC440927 | 0.29 | -1.79 | 0.04 |
| 78988 | MRP63 | 1.92 | 0.94 | 0.02 | 284230 | LOC284230 | 0.29 | -1.79 | 0.02 |
| 9770 | RASSF2 | 1.92 | 0.94 | 0.02 | 3725 | JUN | 0.29 | -1.79 | 0.03 |
| 200030 | LOC200030 | 1.92 | 0.94 | 0.03 | 728553 | LOC728553 | 0.29 | -1.79 | 0.01 |
| 79157 | MFSD11 | 1.92 | 0.94 | 0.00 | 100132564 | LOC100132564 | 0.29 | -1.81 | 0.00 |
| 1316 | KLF6 | 1.91 | 0.94 | 0.04 | 619383 | SCARNA9 | 0.29 | -1.81 | 0.00 |
| 134957 | STXBP5 | 1.90 | 0.93 | 0.02 | 649548 | LOC649548 | 0.28 | -1.81 | 0.00 |
| 8560 | DEGS1 | 1.90 | 0.93 | 0.00 | 114915 | NCRNA00219 | 0.28 | -1.82 | 0.03 |
| 84142 | FAM175A | 1.90 | 0.93 | 0.00 | 391656 | LOC391656 | 0.28 | -1.84 | 0.00 |
| 387 | RHOA | 1.90 | 0.92 | 0.01 | 649076 | LOC649076 | 0.28 | -1.84 | 0.03 |
| 5937 | RBMS1 | 1.89 | 0.92 | 0.00 | 100133662 | LOC100133662 | 0.28 | -1.85 | 0.00 |
| 100130276 | LOC100130276 | 1.89 | 0.92 | 0.00 | 647030 | LOC647030 | 0.28 | -1.85 | 0.04 |
| 64114 | TMBIM1 | 1.89 | 0.92 | 0.01 | 100129553 | LOC100129553 | 0.28 | -1.85 | 0.02 |
| 730060 | LOC730060 | 1.88 | 0.91 | 0.00 | 389342 | LOC389342 | 0.28 | -1.86 | 0.01 |
| 84986 | ARHGAP19 | 1.88 | 0.91 | 0.01 | 646819 | LOC646819 | 0.28 | -1.86 | 0.01 |
| 6874 | TAF4 | 1.88 | 0.91 | 0.03 | 402694 | LOC402694 | 0.27 | -1.87 | 0.00 |
| 147172 | LRRC37B2 | 1.88 | 0.91 | 0.00 | 6194 | RPS6 | 0.27 | -1.87 | 0.03 |
| 56925 | LXN | 1.87 | 0.91 | 0.02 | 9349 | RPL23 | 0.27 | -1.88 | 0.03 |
| 330 | BIRC3 | 1.87 | 0.91 | 0.03 | 728590 | LOC728590 | 0.27 | -1.88 | 0.01 |
| 23390 | ZDHHC17 | 1.87 | 0.91 | 0.02 | 6206 | RPS12 | 0.27 | -1.88 | 0.00 |
| 83734 | ATG10 | 1.87 | 0.90 | 0.01 | 653658 | LOC653658 | 0.27 | -1.88 | 0.03 |
| 144363 | LYRM5 | 1.87 | 0.90 | 0.00 | 6154 | RPL26 | 0.27 | -1.89 | 0.02 |
| 90324 | CCDC97 | 1.86 | 0.89 | 0.00 | 648729 | LOC648729 | 0.27 | -1.89 | 0.00 |
| 54432 | YIPF1 | 1.85 | 0.89 | 0.02 | 6202 | RPS8 | 0.27 | -1.89 | 0.00 |
| 100131718 | LOC100131718 | 1.85 | 0.89 | 0.00 | 647099 | LOC647099 | 0.27 | -1.89 | 0.05 |
| 51279 | C1RL | 1.85 | 0.89 | 0.00 | 100131672 | LOC100131672 | 0.27 | -1.90 | 0.04 |
| 100128288 | LOC100128288 | 1.84 | 0.88 | 0.00 | 1933 | EEF1B2 | 0.27 | -1.90 | 0.00 |
| 5989 | RFX1 | 1.84 | 0.88 | 0.00 | 646294 | LOC646294 | 0.27 | -1.90 | 0.05 |
| 5973 | RENBP | 1.84 | 0.88 | 0.00 | 729903 | LOC729903 | 0.27 | -1.92 | 0.03 |
| 55074 | OXR1 | 1.84 | 0.88 | 0.01 | 144581 | RPL14L | 0.26 | -1.92 | 0.00 |
| 7536 | SF1 | 1.84 | 0.88 | 0.00 | 387066 | SNHG5 | 0.26 | -1.92 | 0.00 |
| 83452 | RAB33B | 1.84 | 0.88 | 0.02 | 649447 | LOC649447 | 0.26 | -1.93 | 0.02 |
| 51439 | FAM8A1 | 1.84 | 0.88 | 0.01 | 730029 | LOC730029 | 0.26 | -1.94 | 0.05 |
| 30 | ACAA1 | 1.84 | 0.88 | 0.03 | 729617 | LOC729617 | 0.26 | -1.95 | 0.05 |
| 150290 | DUSP18 | 1.82 | 0.86 | 0.00 | 728126 | LOC728126 | 0.26 | -1.95 | 0.03 |
| 728105 | LOC728105 | 1.82 | 0.86 | 0.00 | 285053 | LOC285053 | 0.26 | -1.95 | 0.01 |
| 9554 | SEC22B | 1.82 | 0.86 | 0.00 | 644790 | LOC644790 | 0.26 | -1.96 | 0.00 |
| 8766 | RAB11A | 1.81 | 0.85 | 0.01 | 729798 | LOC729798 | 0.26 | -1.97 | 0.04 |
| 10311 | DSCR3 | 1.80 | 0.85 | 0.00 | 6141 | RPL18 | 0.25 | -1.98 | 0.02 |
| 59345 | GNB4 | 1.80 | 0.85 | 0.00 | 646200 | LOC646200 | 0.25 | -1.99 | 0.00 |
| 9605 | C16orf7 | 1.79 | 0.84 | 0.02 | 440589 | LOC440589 | 0.25 | -2.00 | 0.01 |
| 83541 | C20orf55 | 1.79 | 0.84 | 0.01 | 729236 | LOC729236 | 0.25 | -2.01 | 0.00 |
| 79042 | TSEN34 | 1.79 | 0.84 | 0.01 | 285900 | LOC285900 | 0.25 | -2.01 | 0.02 |
| 6789 | STK4 | 1.79 | 0.84 | 0.00 | 731365 | LOC731365 | 0.25 | -2.02 | 0.00 |
| 101 | ADAM8 | 1.79 | 0.84 | 0.01 | 727865 | LOC727865 | 0.24 | -2.05 | 0.00 |
| 6548 | SLC9A1 | 1.78 | 0.83 | 0.02 | 6168 | RPL37A | 0.24 | -2.05 | 0.01 |
| 344787 | ZNF860 | 1.78 | 0.83 | 0.01 | 653079 | LOC653079 | 0.24 | -2.05 | 0.00 |
| 4659 | PPP1R12A | 1.77 | 0.82 | 0.03 | 653737 | LOC653737 | 0.24 | -2.06 | 0.00 |
| 100127975 | LOC100127975 | 1.77 | 0.82 | 0.02 | 390345 | LOC390345 | 0.24 | -2.06 | 0.00 |
| 51635 | DHRS7 | 1.76 | 0.82 | 0.02 | 731640 | LOC731640 | 0.24 | -2.06 | 0.00 |
| 9114 | ATP6V0D1 | 1.76 | 0.82 | 0.05 | 388654 | LOC388654 | 0.24 | -2.06 | 0.02 |
| 64780 | MICAL1 | 1.76 | 0.82 | 0.01 | 347544 | LOC347544 | 0.24 | -2.06 | 0.02 |
| 84061 | MAGT1 | 1.76 | 0.81 | 0.01 | 729362 | LOC729362 | 0.24 | -2.07 | 0.00 |
| 283876 | FLJ39639 | 1.75 | 0.81 | 0.00 | 6152 | RPL24 | 0.24 | -2.07 | 0.01 |
| 84958 | SYTL1 | 1.74 | 0.80 | 0.00 | 6234 | RPS28 | 0.23 | -2.10 | 0.02 |
| 10553 | HTATIP2 | 1.74 | 0.80 | 0.02 | 728782 | LOC728782 | 0.23 | -2.10 | 0.01 |
| 100130516 | LOC100130516 | 1.74 | 0.80 | 0.01 | 6142 | RPL18A | 0.23 | -2.11 | 0.00 |
| 30001 | ERO1L | 1.74 | 0.80 | 0.03 | 100128936 | LOC100128936 | 0.23 | -2.12 | 0.04 |
| 25832 | NBPF14 | 1.73 | 0.79 | 0.01 | 389404 | LOC389404 | 0.23 | -2.13 | 0.00 |
| 10262 | SF3B4 | 1.73 | 0.79 | 0.03 | 644907 | LOC644907 | 0.23 | -2.13 | 0.03 |
| 23041 | MON2 | 1.73 | 0.79 | 0.02 | 6144 | RPL21 | 0.22 | -2.15 | 0.00 |
| 55320 | C14orf106 | 1.73 | 0.79 | 0.03 | 728481 | LOC728481 | 0.22 | -2.17 | 0.02 |
| 10420 | TESK2 | 1.73 | 0.79 | 0.00 | 389156 | LOC389156 | 0.22 | -2.18 | 0.01 |
| 51382 | ATP6V1D | 1.73 | 0.79 | 0.01 | 150094 | SIK1 | 0.22 | -2.18 | 0.02 |
| 222068 | TMED4 | 1.72 | 0.79 | 0.00 | 388532 | LOC388532 | 0.22 | -2.21 | 0.04 |
| 653238 | GTF2H2B | 1.72 | 0.78 | 0.02 | 651436 | LOC651436 | 0.22 | -2.21 | 0.00 |
| 26277 | TINF2 | 1.72 | 0.78 | 0.02 | 651894 | LOC651894 | 0.21 | -2.23 | 0.00 |
| 284161 | GDPD1 | 1.72 | 0.78 | 0.03 | 125144 | C17orf45 | 0.21 | -2.24 | 0.02 |
| 4296 | MAP3K11 | 1.72 | 0.78 | 0.00 | 6192 | RPS4Y1 | 0.21 | -2.24 | 0.05 |
| 9744 | ACAP1 | 1.72 | 0.78 | 0.01 | 29107 | NXT1 | 0.21 | -2.25 | 0.05 |
| 51567 | TTRAP | 1.71 | 0.78 | 0.00 | 645157 | LOC645157 | 0.21 | -2.26 | 0.01 |
| 57700 | KIAA1600 | 1.71 | 0.77 | 0.02 | 641814 | LOC641814 | 0.20 | -2.31 | 0.00 |
| 644474 | LOC644474 | 1.71 | 0.77 | 0.00 | 4736 | RPL10A | 0.20 | -2.34 | 0.01 |
| 55333 | SYNJ2BP | 1.70 | 0.77 | 0.03 | 440055 | LOC440055 | 0.19 | -2.36 | 0.00 |
| 4580 | MTX1 | 1.70 | 0.77 | 0.02 | 390354 | LOC390354 | 0.19 | -2.39 | 0.04 |
| 100129055 | LOC100129055 | 1.70 | 0.77 | 0.03 | 100129028 | LOC100129028 | 0.18 | -2.51 | 0.01 |
| 11033 | CENTA1 | 1.70 | 0.77 | 0.00 | 391370 | LOC391370 | 0.17 | -2.55 | 0.00 |
| 4802 | NFYC | 1.70 | 0.76 | 0.01 | 780851 | SNORD3A | 0.16 | -2.60 | 0.05 |
| 84765 | ZNF577 | 1.69 | 0.76 | 0.04 | 728244 | LOC728244 | 0.16 | -2.62 | 0.04 |
| 54799 | MBTD1 | 1.69 | 0.76 | 0.02 | 762 | CA4 | 0.15 | -2.73 | 0.04 |
| 383 | ARG1 | 1.69 | 0.76 | 0.02 | 338870 | LOC338870 | 0.15 | -2.77 | 0.00 |
| 646547 | LOC646547 | 1.69 | 0.75 | 0.00 | 100129685 | LOC100129685 | 0.14 | -2.80 | 0.02 |
| 126432 | RINL | 1.68 | 0.75 | 0.02 | 653156 | LOC653156 | 0.13 | -2.90 | 0.00 |
| 58986 | TMEM8 | 1.68 | 0.75 | 0.00 |  |  |  |  |  |
| 284390 | ZNF763 | 1.68 | 0.75 | 0.00 |  |  |  |  |  |
| 84078 | KBTBD7 | 1.68 | 0.74 | 0.01 |  |  |  |  |  |
| 9842 | PLEKHM1 | 1.67 | 0.74 | 0.02 |  |  |  |  |  |
| 728070 | LOC728070 | 1.67 | 0.74 | 0.04 |  |  |  |  |  |
| 9637 | FEZ2 | 1.67 | 0.74 | 0.02 |  |  |  |  |  |
| 6667 | SP1 | 1.67 | 0.74 | 0.00 |  |  |  |  |  |
| 951 | CD37 | 1.67 | 0.74 | 0.03 |  |  |  |  |  |
| 9869 | SETDB1 | 1.66 | 0.73 | 0.02 |  |  |  |  |  |
| 100129502 | LOC100129502 | 1.66 | 0.73 | 0.04 |  |  |  |  |  |
| 57238 | KIAA0492 | 1.66 | 0.73 | 0.00 |  |  |  |  |  |
| 5412 | UBL3 | 1.66 | 0.73 | 0.00 |  |  |  |  |  |
| 441087 | LOC441087 | 1.66 | 0.73 | 0.01 |  |  |  |  |  |
| 3295 | HSD17B4 | 1.66 | 0.73 | 0.04 |  |  |  |  |  |
| 3142 | HLX | 1.66 | 0.73 | 0.00 |  |  |  |  |  |
| 3099 | HK2 | 1.66 | 0.73 | 0.02 |  |  |  |  |  |
| 23536 | ADAT1 | 1.66 | 0.73 | 0.03 |  |  |  |  |  |
| 7133 | TNFRSF1B | 1.66 | 0.73 | 0.01 |  |  |  |  |  |
| 10238 | DCAF7 | 1.65 | 0.73 | 0.04 |  |  |  |  |  |
| 57223 | SMEK2 | 1.65 | 0.73 | 0.01 |  |  |  |  |  |
| 55262 | C7orf43 | 1.65 | 0.73 | 0.03 |  |  |  |  |  |
| 10645 | CAMKK2 | 1.65 | 0.72 | 0.00 |  |  |  |  |  |
| 4089 | SMAD4 | 1.65 | 0.72 | 0.02 |  |  |  |  |  |
| 10745 | PHTF1 | 1.64 | 0.72 | 0.01 |  |  |  |  |  |
| 2282 | FKBP1P1 | 1.64 | 0.72 | 0.00 |  |  |  |  |  |
| 7320 | UBE2B | 1.64 | 0.71 | 0.00 |  |  |  |  |  |
| 57827 | C6orf47 | 1.64 | 0.71 | 0.04 |  |  |  |  |  |
| 27334 | P2RY10 | 1.64 | 0.71 | 0.00 |  |  |  |  |  |
| 58528 | RRAGD | 1.64 | 0.71 | 0.01 |  |  |  |  |  |
| 51528 | C14orf100 | 1.63 | 0.70 | 0.00 |  |  |  |  |  |
| 7871 | SLMAP | 1.63 | 0.70 | 0.03 |  |  |  |  |  |
| 7411 | VBP1 | 1.62 | 0.70 | 0.01 |  |  |  |  |  |
| 9325 | TRIP4 | 1.62 | 0.70 | 0.04 |  |  |  |  |  |
| 652755 | LOC652755 | 1.62 | 0.70 | 0.00 |  |  |  |  |  |
| 100133129 | LOC100133129 | 1.62 | 0.70 | 0.01 |  |  |  |  |  |
| 55508 | SLC35E3 | 1.62 | 0.69 | 0.00 |  |  |  |  |  |
| 100132391 | LOC100132391 | 1.62 | 0.69 | 0.03 |  |  |  |  |  |
| 54971 | BANP | 1.62 | 0.69 | 0.00 |  |  |  |  |  |
| 284757 | LOC284757 | 1.62 | 0.69 | 0.00 |  |  |  |  |  |
| 250 | ALPP | 1.61 | 0.69 | 0.02 |  |  |  |  |  |
| 80019 | UBTD1 | 1.61 | 0.68 | 0.00 |  |  |  |  |  |
| 55276 | PGM2 | 1.61 | 0.68 | 0.03 |  |  |  |  |  |
| 55012 | PPP2R3C | 1.61 | 0.68 | 0.02 |  |  |  |  |  |
| 7317 | UBE1 | 1.61 | 0.68 | 0.04 |  |  |  |  |  |
| 2591 | GALNT3 | 1.61 | 0.68 | 0.04 |  |  |  |  |  |
| 7568 | ZNF20 | 1.60 | 0.68 | 0.02 |  |  |  |  |  |
| 1455 | CSNK1G2 | 1.60 | 0.68 | 0.03 |  |  |  |  |  |
| 200315 | APOBEC3A | 1.60 | 0.68 | 0.01 |  |  |  |  |  |
| 51259 | TMEM216 | 1.60 | 0.68 | 0.00 |  |  |  |  |  |
| 128387 | TATDN3 | 1.60 | 0.68 | 0.03 |  |  |  |  |  |
| 10628 | TXNIP | 1.60 | 0.68 | 0.03 |  |  |  |  |  |
| 23240 | KIAA0922 | 1.60 | 0.67 | 0.00 |  |  |  |  |  |
| 9896 | FIG4 | 1.59 | 0.67 | 0.04 |  |  |  |  |  |
| 9778 | KIAA0232 | 1.59 | 0.67 | 0.04 |  |  |  |  |  |
| 4664 | NAB1 | 1.59 | 0.67 | 0.02 |  |  |  |  |  |
| 53635 | PTOV1 | 1.59 | 0.67 | 0.00 |  |  |  |  |  |
| 8876 | VNN1 | 1.59 | 0.67 | 0.04 |  |  |  |  |  |
| 89970 | RSPRY1 | 1.58 | 0.66 | 0.00 |  |  |  |  |  |
| 255967 | PAN3 | 1.58 | 0.66 | 0.03 |  |  |  |  |  |
| 3344 | FOXN2 | 1.58 | 0.66 | 0.04 |  |  |  |  |  |
| 64395 | GMCL1 | 1.58 | 0.66 | 0.01 |  |  |  |  |  |
| 154881 | KCTD7 | 1.58 | 0.66 | 0.02 |  |  |  |  |  |
| 9683 | N4BP1 | 1.58 | 0.66 | 0.03 |  |  |  |  |  |
| 3385 | ICAM3 | 1.58 | 0.66 | 0.03 |  |  |  |  |  |
| 202243 | CCDC125 | 1.58 | 0.66 | 0.05 |  |  |  |  |  |
| 399761 | BMS1P5 | 1.57 | 0.65 | 0.04 |  |  |  |  |  |
| 100128507 | LOC100128507 | 1.57 | 0.65 | 0.00 |  |  |  |  |  |
| 9966 | TNFSF15 | 1.57 | 0.65 | 0.03 |  |  |  |  |  |
| 10402 | ST3GAL6 | 1.57 | 0.65 | 0.02 |  |  |  |  |  |
| 10299 | MARCH6 | 1.56 | 0.64 | 0.01 |  |  |  |  |  |
| 285696 | FLJ34047 | 1.56 | 0.64 | 0.02 |  |  |  |  |  |
| 148203 | ZNF738 | 1.56 | 0.64 | 0.04 |  |  |  |  |  |
| 729659 | LOC729659 | 1.56 | 0.64 | 0.00 |  |  |  |  |  |
| 8621 | CDC2L5 | 1.56 | 0.64 | 0.02 |  |  |  |  |  |
| 100129362 | LOC100129362 | 1.55 | 0.64 | 0.04 |  |  |  |  |  |
| 1845 | DUSP3 | 1.55 | 0.64 | 0.00 |  |  |  |  |  |
| 100129518 | LOC100129518 | 1.55 | 0.63 | 0.04 |  |  |  |  |  |
| 10093 | ARPC4 | 1.55 | 0.63 | 0.02 |  |  |  |  |  |
| 23295 | MGRN1 | 1.55 | 0.63 | 0.04 |  |  |  |  |  |
| 8705 | B3GALT4 | 1.54 | 0.63 | 0.01 |  |  |  |  |  |
| 23625 | FAM89B | 1.54 | 0.63 | 0.03 |  |  |  |  |  |
| 339344 | MYPOP | 1.54 | 0.63 | 0.00 |  |  |  |  |  |
| 4650 | MYO9B | 1.54 | 0.63 | 0.00 |  |  |  |  |  |
| 9849 | ZNF518A | 1.54 | 0.63 | 0.02 |  |  |  |  |  |
| 83861 | RSPH3 | 1.54 | 0.62 | 0.03 |  |  |  |  |  |
| 162466 | PHOSPHO1 | 1.54 | 0.62 | 0.02 |  |  |  |  |  |
| 406991 | MIR21 | 1.54 | 0.62 | 0.01 |  |  |  |  |  |
| 1050 | CEBPA | 1.53 | 0.62 | 0.03 |  |  |  |  |  |
| 6084 | RNY1 | 1.53 | 0.61 | 0.00 |  |  |  |  |  |
| 1820 | ARID3A | 1.53 | 0.61 | 0.04 |  |  |  |  |  |
| 79958 | DENND1C | 1.53 | 0.61 | 0.00 |  |  |  |  |  |
| 79903 | NAT15 | 1.52 | 0.61 | 0.00 |  |  |  |  |  |
| 3087 | HHEX | 1.52 | 0.60 | 0.03 |  |  |  |  |  |
| 51765 | MST4 | 1.52 | 0.60 | 0.04 |  |  |  |  |  |
| 10559 | SLC35A1 | 1.51 | 0.60 | 0.02 |  |  |  |  |  |
| 129531 | MITD1 | 1.51 | 0.59 | 0.03 |  |  |  |  |  |
| 7351 | UCP2 | 1.51 | 0.59 | 0.02 |  |  |  |  |  |
| 1432 | MAPK14 | 1.51 | 0.59 | 0.01 |  |  |  |  |  |
| 8312 | AXIN1 | 1.51 | 0.59 | 0.04 |  |  |  |  |  |
| 10162 | LPCAT3 | 1.51 | 0.59 | 0.04 |  |  |  |  |  |
| 5860 | QDPR | 1.51 | 0.59 | 0.00 |  |  |  |  |  |
| 10565 | ARFGEF1 | 1.50 | 0.59 | 0.02 |  |  |  |  |  |
| 57658 | CALCOCO1 | 1.50 | 0.59 | 0.02 |  |  |  |  |  |
| 4801 | NFYB | 1.50 | 0.59 | 0.00 |  |  |  |  |  |
| 5732 | PTGER2 | 1.50 | 0.58 | 0.02 |  |  |  |  |  |
| 10210 | TOPORS | 1.50 | 0.58 | 0.00 |  |  |  |  |  |
| 80124 | VCPIP1 | 1.50 | 0.58 | 0.02 |  |  |  |  |  |
| 116985 | ARAP1 | 1.50 | 0.58 | 0.02 |  |  |  |  |  |
| 285754 | FLJ37396 | 1.50 | 0.58 | 0.03 |  |  |  |  |  |
| 129642 | MBOAT2 | 1.49 | 0.58 | 0.02 |  |  |  |  |  |
| 29110 | TBK1 | 1.49 | 0.58 | 0.05 |  |  |  |  |  |
| 55251 | PCMTD2 | 1.49 | 0.57 | 0.04 |  |  |  |  |  |
| 9993 | DGCR2 | 1.49 | 0.57 | 0.04 |  |  |  |  |  |
| 9546 | APBA3 | 1.49 | 0.57 | 0.00 |  |  |  |  |  |
| 81 | ACTN4 | 1.48 | 0.57 | 0.00 |  |  |  |  |  |
| 83641 | FAM107B | 1.48 | 0.57 | 0.05 |  |  |  |  |  |
| 79730 | NSUN7 | 1.48 | 0.57 | 0.01 |  |  |  |  |  |
| 83463 | MXD3 | 1.48 | 0.57 | 0.00 |  |  |  |  |  |
| 54925 | ZNF434 | 1.48 | 0.57 | 0.05 |  |  |  |  |  |
| 11284 | PNKP | 1.48 | 0.57 | 0.01 |  |  |  |  |  |
| 161253 | REM2 | 1.48 | 0.56 | 0.04 |  |  |  |  |  |
| 648059 | LOC648059 | 1.48 | 0.56 | 0.00 |  |  |  |  |  |
| 11193 | WBP4 | 1.48 | 0.56 | 0.05 |  |  |  |  |  |
| 10238 | WDR68 | 1.48 | 0.56 | 0.02 |  |  |  |  |  |
| 26097 | C1orf77 | 1.47 | 0.56 | 0.01 |  |  |  |  |  |
| 23032 | USP33 | 1.47 | 0.56 | 0.00 |  |  |  |  |  |
| 27141 | CIDEB | 1.47 | 0.56 | 0.05 |  |  |  |  |  |
| 9600 | PITPNM1 | 1.47 | 0.55 | 0.02 |  |  |  |  |  |
| 112616 | CMTM7 | 1.47 | 0.55 | 0.04 |  |  |  |  |  |
| 7702 | ZNF143 | 1.47 | 0.55 | 0.05 |  |  |  |  |  |
| 90120 | C9orf69 | 1.46 | 0.55 | 0.03 |  |  |  |  |  |
| 3305 | HSPA1L | 1.46 | 0.55 | 0.02 |  |  |  |  |  |
| 23592 | LEMD3 | 1.46 | 0.54 | 0.01 |  |  |  |  |  |
| 255809 | LOC255809 | 1.46 | 0.54 | 0.02 |  |  |  |  |  |
| 55762 | ZNF701 | 1.46 | 0.54 | 0.00 |  |  |  |  |  |
| 5165 | PDK3 | 1.45 | 0.54 | 0.03 |  |  |  |  |  |
| 84961 | FBXL20 | 1.45 | 0.54 | 0.00 |  |  |  |  |  |
| 2011 | MARK2 | 1.45 | 0.54 | 0.03 |  |  |  |  |  |
| 728417 | LOC728417 | 1.45 | 0.53 | 0.04 |  |  |  |  |  |
| 374907 | B3GNT8 | 1.45 | 0.53 | 0.00 |  |  |  |  |  |
| 100133840 | LOC100133840 | 1.44 | 0.53 | 0.05 |  |  |  |  |  |
| 80820 | EEPD1 | 1.44 | 0.52 | 0.00 |  |  |  |  |  |
| 641710 | LOC641710 | 1.44 | 0.52 | 0.01 |  |  |  |  |  |
| 10636 | RGS14 | 1.43 | 0.52 | 0.03 |  |  |  |  |  |
| 100132119 | LOC100132119 | 1.43 | 0.52 | 0.02 |  |  |  |  |  |
| 64400 | AKTIP | 1.42 | 0.51 | 0.04 |  |  |  |  |  |
| 646897 | LOC646897 | 1.42 | 0.51 | 0.01 |  |  |  |  |  |
| 2588 | GALNS | 1.42 | 0.51 | 0.03 |  |  |  |  |  |
| 343990 | C2orf55 | 1.42 | 0.50 | 0.03 |  |  |  |  |  |
| 408 | ARRB1 | 1.42 | 0.50 | 0.04 |  |  |  |  |  |
| 60684 | C4orf41 | 1.42 | 0.50 | 0.05 |  |  |  |  |  |
| 646463 | LOC646463 | 1.41 | 0.50 | 0.02 |  |  |  |  |  |
| 23199 | KIAA0182 | 1.41 | 0.50 | 0.01 |  |  |  |  |  |
| 6484 | ST3GAL4 | 1.41 | 0.50 | 0.02 |  |  |  |  |  |
| 1523 | CUTL1 | 1.41 | 0.50 | 0.02 |  |  |  |  |  |
| 79894 | ZNF672 | 1.41 | 0.49 | 0.03 |  |  |  |  |  |
| 5518 | PPP2R1A | 1.41 | 0.49 | 0.02 |  |  |  |  |  |
| 153830 | RNF145 | 1.41 | 0.49 | 0.03 |  |  |  |  |  |
| 10126 | DNAL4 | 1.41 | 0.49 | 0.03 |  |  |  |  |  |
| 5298 | PI4KB | 1.41 | 0.49 | 0.03 |  |  |  |  |  |
| 5990 | RFX2 | 1.40 | 0.49 | 0.05 |  |  |  |  |  |
| 51314 | TXNDC3 | 1.39 | 0.48 | 0.02 |  |  |  |  |  |
| 64748 | LPPR2 | 1.39 | 0.48 | 0.03 |  |  |  |  |  |
| 489 | ATP2A3 | 1.38 | 0.47 | 0.01 |  |  |  |  |  |
| 375260 | MGC52000 | 1.38 | 0.47 | 0.02 |  |  |  |  |  |
| 9968 | MED12 | 1.38 | 0.47 | 0.01 |  |  |  |  |  |
| 319 | APOF | 1.38 | 0.47 | 0.02 |  |  |  |  |  |
| 8260 | ARD1A | 1.38 | 0.46 | 0.02 |  |  |  |  |  |
| 91289 | LMF2 | 1.38 | 0.46 | 0.03 |  |  |  |  |  |
| 8794 | TNFRSF10C | 1.38 | 0.46 | 0.04 |  |  |  |  |  |
| 3148 | HMGB2 | 1.37 | 0.46 | 0.04 |  |  |  |  |  |
| 29933 | GPR132 | 1.37 | 0.46 | 0.01 |  |  |  |  |  |
| 128989 | C22orf25 | 1.37 | 0.46 | 0.01 |  |  |  |  |  |
| 11054 | OGFR | 1.37 | 0.46 | 0.04 |  |  |  |  |  |
| 54877 | ZCCHC2 | 1.36 | 0.45 | 0.03 |  |  |  |  |  |
| 100131786 | LOC100131786 | 1.36 | 0.44 | 0.04 |  |  |  |  |  |
| 10135 | PBEF1 | 1.36 | 0.44 | 0.01 |  |  |  |  |  |
| 6251 | RSU1 | 1.35 | 0.44 | 0.01 |  |  |  |  |  |
| 6478 | SIAH2 | 1.35 | 0.43 | 0.04 |  |  |  |  |  |
| 285296 | LOC285296 | 1.35 | 0.43 | 0.04 |  |  |  |  |  |
| 55884 | WSB2 | 1.35 | 0.43 | 0.04 |  |  |  |  |  |
| 8694 | DGAT1 | 1.35 | 0.43 | 0.02 |  |  |  |  |  |
| 54480 | CHPF2 | 1.34 | 0.43 | 0.02 |  |  |  |  |  |
| 317662 | FAM149B1 | 1.34 | 0.42 | 0.04 |  |  |  |  |  |
| 93556 | C3orf50 | 1.34 | 0.42 | 0.04 |  |  |  |  |  |
| 89941 | RHOT2 | 1.34 | 0.42 | 0.05 |  |  |  |  |  |
| 642788 | LOC642788 | 1.34 | 0.42 | 0.01 |  |  |  |  |  |
| 55069 | C7orf42 | 1.34 | 0.42 | 0.04 |  |  |  |  |  |
| 80228 | ORAI2 | 1.33 | 0.42 | 0.02 |  |  |  |  |  |
| 728790 | LOC728790 | 1.33 | 0.41 | 0.04 |  |  |  |  |  |
| 146540 | ZNF785 | 1.33 | 0.41 | 0.04 |  |  |  |  |  |
| 25816 | TNFAIP8 | 1.33 | 0.41 | 0.01 |  |  |  |  |  |
| 653043 | LOC653043 | 1.33 | 0.41 | 0.03 |  |  |  |  |  |
| 100128591 | LOC100128591 | 1.33 | 0.41 | 0.02 |  |  |  |  |  |
| 55690 | PACS1 | 1.32 | 0.41 | 0.03 |  |  |  |  |  |
| 23609 | MKRN2 | 1.32 | 0.41 | 0.05 |  |  |  |  |  |
| 401620 | LOC401620 | 1.32 | 0.40 | 0.02 |  |  |  |  |  |
| 389677 | RBM12B | 1.32 | 0.40 | 0.02 |  |  |  |  |  |
| 100130952 | LOC100130952 | 1.31 | 0.39 | 0.03 |  |  |  |  |  |
| 201294 | UNC13D | 1.31 | 0.39 | 0.04 |  |  |  |  |  |
| 728895 | LOC728895 | 1.30 | 0.38 | 0.03 |  |  |  |  |  |
| 11033 | ADAP1 | 1.30 | 0.37 | 0.03 |  |  |  |  |  |
| 22931 | RAB18 | 1.28 | 0.36 | 0.05 |  |  |  |  |  |
| 94097 | SFXN5 | 1.28 | 0.36 | 0.03 |  |  |  |  |  |
| 5595 | MAPK3 | 1.27 | 0.35 | 0.02 |  |  |  |  |  |
| 2885 | GRB2 | 1.27 | 0.34 | 0.03 |  |  |  |  |  |
| 5429 | POLH | 1.24 | 0.31 | 0.03 |  |  |  |  |  |
| 8740 | TNFSF14 | 1.17 | 0.23 | 0.01 |  |  |  |  |  |

**Supplementary Table 2**

**Genes progressively upregulated or downregulated in MGUS- and MM-HDN compared to healthy subjects**

| **ENTREZ**  **GENE_ID** | **SYMBOL** | **ctrl.AVG**  **Signal** | **MGUS.AVG**  **Signal** | **MM.AVG**  **Signal** | **MGUS vs CTRL**  **Fold change** | **MGUS vs CTRL**  **p-value** | **MM vs CTRL**  **Fold change** | **MM vs CTRL**  **p-value** | **MGUS vs MM**  **Fold change** | **MGUS vs MM**  **p-value** |
| --- | --- | --- | --- | --- | --- | --- | --- | --- | --- | --- |
| **1445** | **CSK** | 274.6 | 451.8 | 1120.5 | 1.65 | 0.00 | 4.08 | 0.02 | 2.48 | 0.05 |
| **25801** | **GCA** | 821 | 1764.2 | 5434.5 | 2.15 | 0.00 | 6.62 | 0.00 | 3.08 | 0.01 |
| **1955** | **MEGF9** | 132.4 | 236.5 | 389.4 | 1.79 | 0.04 | 2.94 | 0.03 | 1.65 | 0.04 |
| **5236** | **PGM1** | 183.2 | 295.3 | 450.5 | 1.61 | 0.00 | 2.46 | 0.00 | 1.53 | 0.01 |
| **60675** | **PROK2** | 915.2 | 2393.2 | 6236.2 | 2.61 | 0.01 | 6.81 | 0.00 | 2.61 | 0.00 |
| **51759** | **C9orf78** | 1032.4 | 618.5 | 431.9 | 0.60 | 0.02 | 0.42 | 0.00 | 0.70 | 0.01 |
| **2483** | **FRG1** | 531.3 | 327.3 | 232.9 | 0.62 | 0.02 | 0.44 | 0.00 | 0.71 | 0.01 |
| **9929** | **JOSD1** | 574 | 276.2 | 188.9 | 0.48 | 0.05 | 0.33 | 0.00 | 0.68 | 0.00 |

**Supplementary Table 3**

**Enriched STAT- genes-associated to CD64, significantly downregulated in MGUS-HDN compared to MM-HDN**

| **GENE SYMBOL** | **GENE_TITLE** | **Fold change** | **p-value** |
| --- | --- | --- | --- |
| *Enriched STAT-3 genes associated to CD64, significantly downregulated in MGUS-HDN compared to MM-HDN* | | | |
| ***FCGR1A*** | Fc fragment of IgG, high affinity Ia, receptor (CD64) | -0.65 | <0.0001 |
| ***IFNAR2*** | interferon (alpha, beta and omega) receptor 2 | -0.40 | <0.0001 |
| ***JAK2*** | Janus kinase 2 (a protein tyrosine kinase) | -0.63 | <0.0001 |
| ***STAT3*** | signal transducer and activator of transcription 3 (acute-phase response factor) | -0.29 | <0.0001 |
| ***SOCS3*** | suppressor of cytokine signaling 3 | -0.56 | <0.0001 |
| ***SERPING1*** | serpin peptidase inhibitor, clade G (C1 inhibitor), member 1, (angioedema, hereditary) | -0.54 | <0.0001 |
| ***IRF1*** | interferon regulatory factor 1 | -0.15 | <0.0001 |
| ***TNFRSF1A*** | TNF receptor superfamily member 1A | -0.26 | 0.02 |
| ***TNFRSF1B*** | TNF receptor superfamily member 1B | -0.15 | <0.0001 |
| *Enriched STAT-6 genes associated to CD64, significantly downregulated in MGUS-HDN compared to MM-HDN* | | | |
| ***SOCS1*** | suppressor of cytokine signaling 1 | -0.60 | <0.0001 |
| ***PARP12*** | poly (ADP-ribose) polymerase family, member 12 | -0.60 | <0.0001 |
| ***IRF2*** | interferon regulatory factor 2 | -0.58 | <0.0001 |
| ***PARP14*** | poly (ADP-ribose) polymerase family, member 14 | -0.45 | <0.0001 |
| ***IRF7*** | interferon regulatory factor 7 | -0.43 | <0.0001 |
| ***IL4R*** | interleukin 4 receptor | -0.42 | <0.0001 |
| ***NFKBIA*** | nuclear factor of kappa light polypeptide gene enhancer in B-cells inhibitor, alpha | -0.65 | <0.0001 |
| *Enriched STAT-5a genes associated to CD64, significantly downregulated in MGUS-HDN compared to MM-HDN* | | | |
| ***SOCS3*** | suppressor of cytokine signaling 3 | -0.60 | <0.0001 |

**Supplementary Table 4**

**Patients characteristics of 60 MM and 30 MGUS patients participating to the study, including 47 newly-diagnosed MM candidates to VTD induction regimen**

| **Characteristics** | **Newly-diagnosed MM (N=60)** | | **MGUS** |
| --- | --- | --- | --- |
|  | **Candidates to VTD regimen,**  **N=47** | **Not candidates to VTD regimen,**  **N=13** | **N=30** |
| **Median age (range)** | 64 (54-68) | 68 (66-75) | 63 (45-79) |
| **Males/Females, n** | 30/13 (70/30) | 9/4 (69/31) | 20/10 (75/25) |
| **Serum isotype** |  |  |  |
| *IgG, n (%)* | 25 (58) | 8 (62) | 22 (73) |
| *IgA, n (%)* | 9 (21) | 4 (31) | 8 (27) |
| *Micromolecular, n (%)* | 6 (14) | 1(7) | 0 (0) |
| *Non-secretory, n (%)* | 3 (7) | 0 (0) | 0 (0) |
| **ISS** |  |  |  |
| *I, n (%)* | 11 (26) | 3 (22) |  |
| *II, n (%)* | 13 (30) | 8 (63) | NA |
| *III, n (%)* | 19 (44) | 2(15) |  |
| **FISH analysis available** | **32 (68.1)** | **5 (39)** | **6 (20)** |
| *Standard Risk, n (%)* | 21 (49) | 5 (39) | 6 (20) |
| *High risk, n (%)* | 11 (25.5) | 0 (0) | 0 (0) |
| **> 3 bone lesions, n (%)** | 27 (63) | 10 (77) | 0 (0) |
| **Clearance creatinine<30 ml/min, n (%)** | 5 (12) | 3 (22) | 0 (0) |
| **Extramedullar disease, n (%)** | 6 (14) | 1 (7) | 0 (0) |
